# Adaptive Hydrogenation of Alkynes Using CO as a Molecular Trigger to Selectively Produce Alkanes or *Z*‐Alkenes

**DOI:** 10.1002/anie.9302519

**Published:** 2026-08-06

**Authors:** Manisha Durai, Lachlan Sharp‐Bucknall, Tim Alexander Schubert, Jacob Johny, John‐Tommes Krzeslack, Walid Hetaba, Walter Leitner, Alexis Bordet

**Affiliations:** ^1^ Max Planck Institute For Chemical Energy Conversion Mülheim an der Ruhr Germany; ^2^ Institute For Technical and Macromolecular Chemistry RWTH Aachen University Aachen Germany

**Keywords:** adaptive hydrogenation, carbon monoxide, chemoselectivity, molecular triggers, palladium catalysis

## Abstract

The selective hydrogenation of alkynes to either alkanes or alkenes is an important step in synthetic processes across the entire chemical value chain with a broad range of applications especially for fine chemical and pharmaceutical production. While traditional developments aim at individual catalysts optimized for either one or the other product, catalytic systems capable of adaptively targeting both classes of products with high activity and selectivity could enable flexible production schemes. Here, we show that CO can be used as a molecular trigger to dynamically adjust the selectivity of supported palladium nanoparticles (NPs) in alkyne hydrogenation. In particular, Pd NPs immobilized on an imidazolium‐based supported ionic liquid phase (Pd@SILP) hydrogenate a wide range of structurally diverse alkynes, delivering synthetically relevant alkane or *Z*‐alkene products under H_2_ or H_2_/CO as feed gas, respectively. Reference experiments, kinetic studies including isotope labeling, and near‐ambient‐pressure XPS studies reveal that the rapid and robust selectivity switch originates from the reversible adsorption of CO competing with alkene at the Pd surface. In contrast to its notorious reputation as a catalyst poison in hydrogenation, these findings establish CO as an effective molecular trigger for adaptive catalysis, paving the way toward even broader applications for reversible selectivity control.

## Graphical abstract

One catalyst for two products: CO acts as a molecular trigger for adaptive alkyne hydrogenation using supported Pd nanoparticles. The catalyst reversibly switches between the formation of alkanes under H_2_ and *Z*‐alkenes under H_2_/CO for 37 structurally diverse alkynes. Redefining the role of CO in metal‐catalyzed hydrogenation, this method offers broad functional‐group tolerance and access to valuable bioactive and pharmaceutical intermediates.

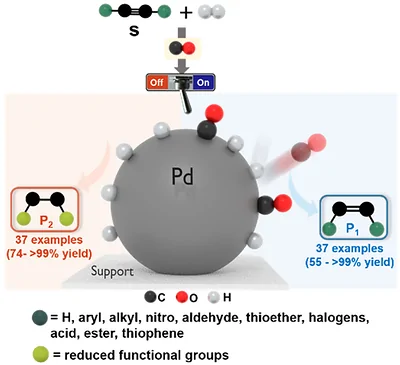

## 1 Introduction

The development of catalytic systems that can be dynamically adjusted or even self‐adjust to different states of performance is a field of growing interest for innovative chemical production schemes [1, 2, 3, 4, 5]. The concept is particularly attractive for divergent reactions that can lead to different valuable products from the same starting material [6, 7]. In this context, the selective hydrogenation of alkynes to either alkanes or alkenes stands as a cornerstone transformation in modern synthetic chemistry, enabling the construction of high‐value intermediates central to pharmaceuticals, agrochemicals, and fine chemicals [8, 9]. Achieving precise control over chemo‐ and stereoselectivity in alkyne hydrogenation remains a persistent challenge, particularly for multifunctional alkynes containing a wide range of sensitive and potentially reducible functionalities (e.g., –NO_2_, –CHO, –C═O, –Cl, –Br, –S) [10, 11]. Conventional approaches using Lindlar's catalyst (Pd/CaCO_3_ poisoned with Pb and quinoline) or Birch reduction to access alkene products are hampered by the use of toxic additives, poor recyclability, and poor functional group tolerance [12, 13]. Catalysis research witnessed important advances in this field in the past decades, in particular through the development of strategies involving bimetallic [14, 15] and single‐atom [16, 17] structures, catalysts surface modification by ligands [18, 19, 20], poisons [21, 22, 23, 24, 25], and ionic liquids [26], and careful tuning of the reaction environment [26, 27, 28, 29]. However, all catalysts reported so far were designed for static performance under optimized reaction conditions to maximize the yield of either the alkane or alkene products individually (Figure 1a). The dynamic and adaptive control of such selectivities with a single catalytic system has not been achieved up to now.

**FIGURE 1:**
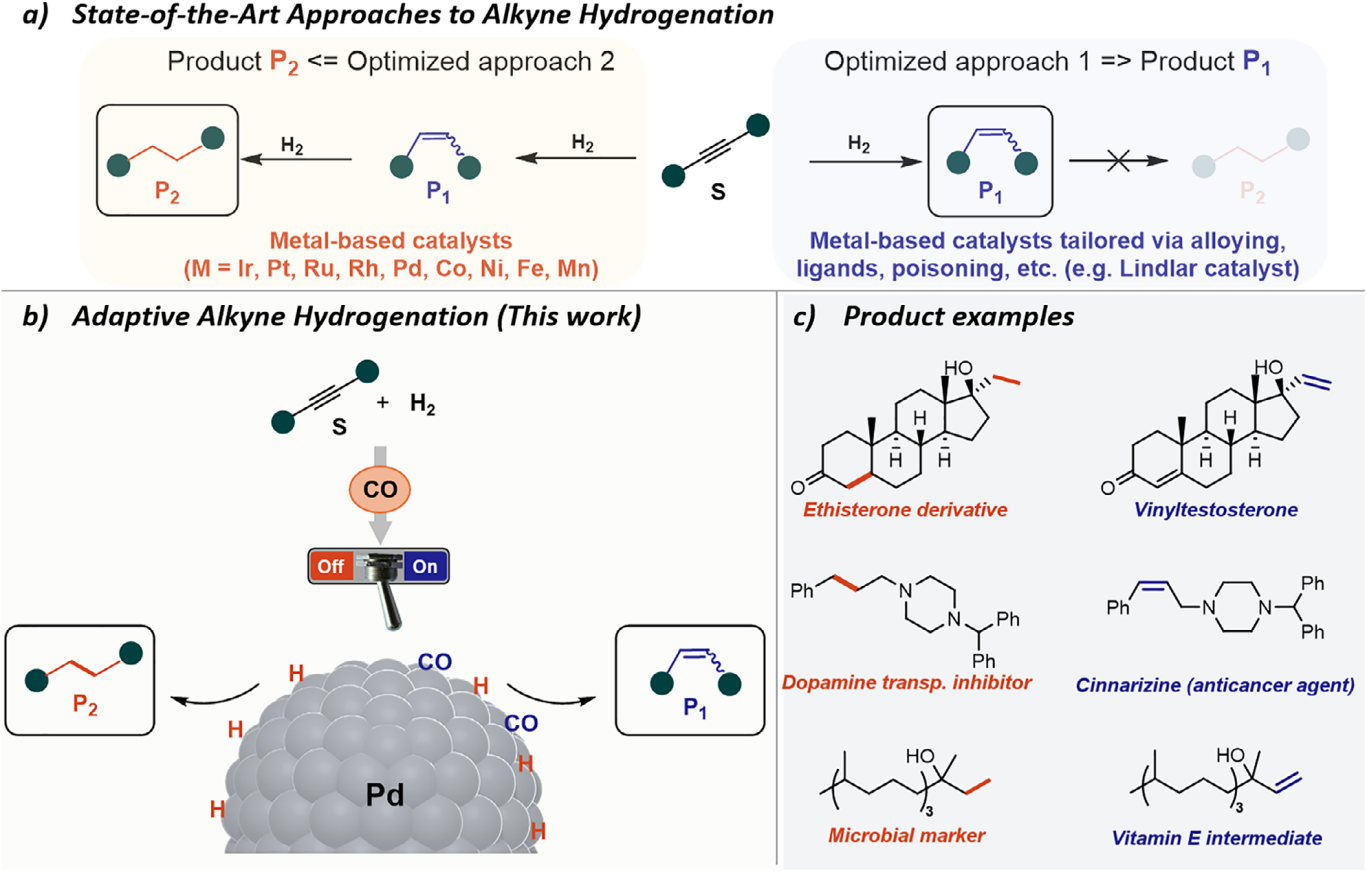
(a) Typical approaches to alkyne hydrogenation using individual optimized catalysts and conditions to produce either alkenes (P1) or alkanes (P2). (b) Our approach to adaptive alkyne hydrogenation using CO as a molecular trigger and access both product classes over the same Pd‐catalyst by simply switching the feed gas. (c) Selected examples of bio‐active alkenes and alkanes product couples produced with this adaptive system.

Molecular triggers are particularly attractive for dynamic control of catalyst performance as they can induce rapid and reversible changes through chemical interactions with the active centers [1]. Recent studies from our team showed that CO_2_ can act as such a trigger, modulating hydrogenation selectivity via reversible generation of formate species at the catalyst surface competing with substrate adsorption [30, 31, 32]. In the search for other small molecules that are known to modify catalyst surfaces, we extended our interest to carbon monoxide (CO), which is known as a strong ligand and catalyst poison [33, 34, 35] that has also been used as a selectivity regulator in hydrogenation [36, 37]. In particular, prior work by Pérez‐Ramírez highlighted that surface modification of Pd‐catalysts with CO can enhance the selectivity in the hydrogenation of acetylene to ethene and other simple alkynes [38, 39]. In a recent study, we demonstrated CO‐mediated selective hydrogenation of ynones to *E*‐chalcone derivatives using a multifunctional Ru@SiO_2_‐[Pd‐NHC] catalyst [40]. Since the poisoning properties of CO for metal surfaces are well‐known, the strong interactions would not be expected to be reversible under standard conditions and have consequently not been exploited in switchable or adaptive systems up to now.

Challenging this presumption, we hypothesized that CO might be able to act as a molecular trigger to control the reactivity of supported metal nanoparticles (NPs) in hydrogenation in a fully reversible, rapid, and robust manner. In this work, we validate this concept for supported Pd NP catalysts in the adaptive hydrogenation of a broad range of functionalized alkynes (Figure 1b). The practical potential is demonstrated for synthetically relevant examples where simply applying either H_2_ of H_2_/CO as the feed gas switches reversibly between the production of either alkanes or alkenes with high selectivity (Figure 1c).

## 2 Results and Discussion

### 2.1 Catalyst Preparation and Characterization

A series of commercially available and custom synthesized supported Pd NPs was chosen as benchmark catalysts to explore the concept. In addition to standard materials, amorphous silica modified with ionic liquid‐type molecular structures (SILP) was used as support. This SILP was selected for its proven suitability as a stabilizing matrix for synthesizing and stabilizing various mono‐ and bimetallic NPs used for hydrogenation and hydrodeoxygenation reactions [41]. The Pd@SILP catalyst was synthesized following a procedure adapted from our previous work [42]. Briefly, the SILP was prepared via silanization of the imidazolium‐based ionic liquid [1‐butyl‐3‐(3‐triethoxy‐silylpropyl)‐imidazolium]NTf_2_ (IL) on dehydroxylated (500°C under vacuum, 18 h) amorphous SiO_2_. A Brunauer–Emmett–Teller (BET) surface area of 239 m^2^ g^−1^ was measured, substantially lower than that of the initial SiO_2_ (332 m^2^ g^−1^), and the Si─O─Si bonds between the SiO_2_ surface and the IL‐like structure were confirmed by ^29^Si solid‐state NMR (Table S1) [43]. The N1s x‐ray photoelectron spectroscopy (XPS) spectrum shows a component at ∼401.7 eV characteristic of the imidazolium cation (Figure 2c) [44]. The second N1s component at ∼399.5 eV and the F1s signal at ∼689.0 eV are characteristic of the bis(trifluoromethylsulfonyl)imide (NTf_2_^−^) anion (Figure 2d) [45]. Palladium NPs were deposited on the SILP by decomposition of a mesitylene solution of [Pd_2_(dba)_3_] (dba = dibenzylideneacetone) under a hydrogen atmosphere (3 bar) at 150 °C (Figure 2a, and see the Supporting Information for experimental details).

**FIGURE 2:**
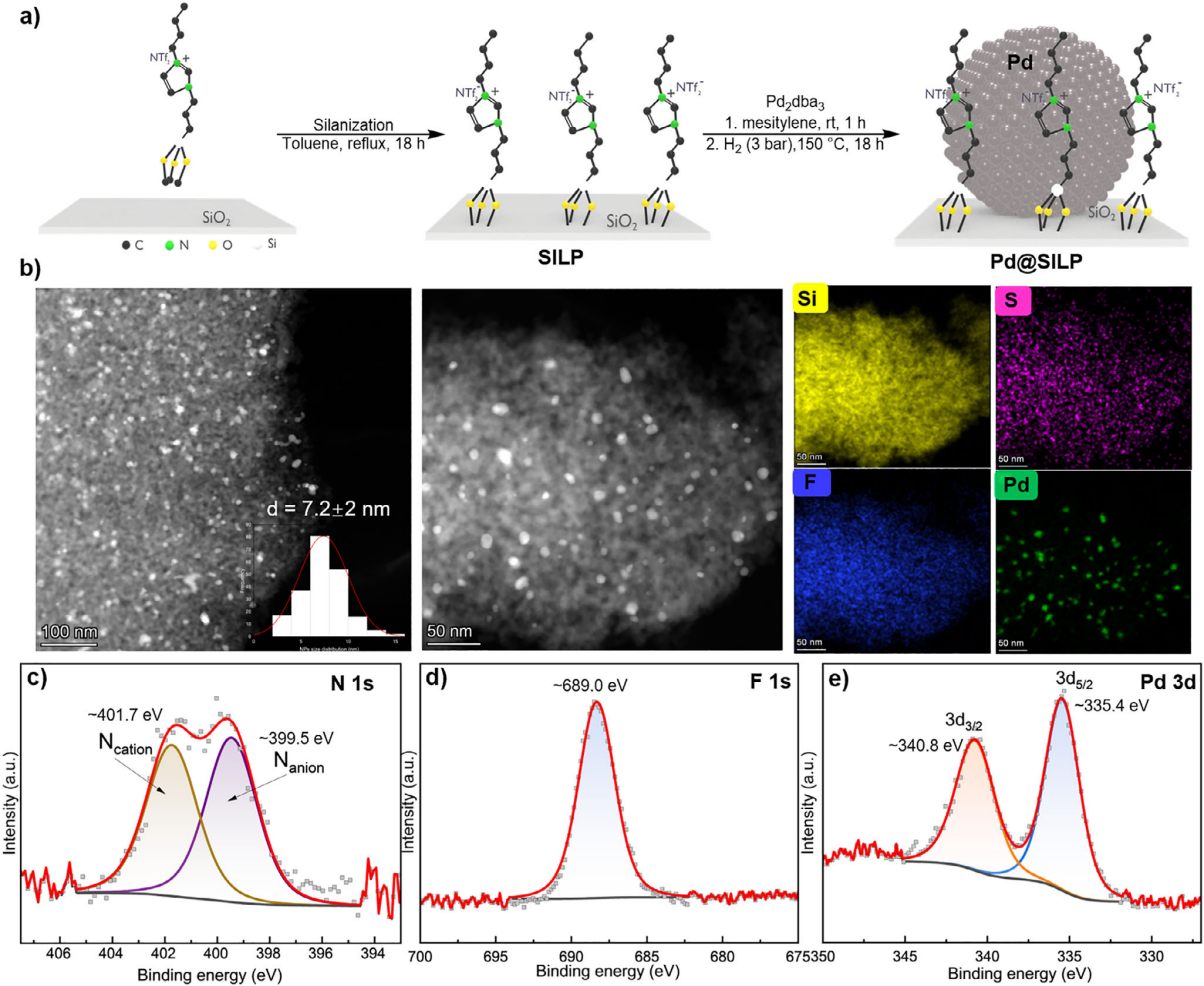
Synthesis and characterization of Pd@SILP. (a) Scheme illustrating the two‐step preparation of Pd@SILP, and its characterization by (b) HAADF‐STEM images (100 and 50 nm scales) and EDX elemental mapping (50 nm scales) of Si‐Kα; S‐ Kα; F‐ Kα; Pd‐Lα; and high‐resolution XPS spectra of (c) N1s, (d) F1s and (e) Pd3d.

Inductively coupled plasma optical emission spectroscopy (ICP‐OES) analysis revealed a Pd loading of 1.99 wt% (Table S1), in excellent agreement with the theoretical value of 2 wt%. High‐angle annular dark‐field scanning transmission electron microscopy (HAADF‐STEM) imaging confirmed the formation of well‐dispersed Pd NPs with an average diameter of 7.2 ± 2.0 nm (Figures 2b and S1). The Pd3d XPS spectrum (Figure 2e) shows signals at ∼335.4 eV (Pd3d5/2) and ∼340.8 eV (Pd3d3/2) which are consistent with the literature values for Pd(0) [46], indicating that the Pd NPs are in a metallic state.

### 2.2 Catalytic Study

The hydrogenation of 4‐phenylbut‐3‐yn‐2‐one (**1**) was selected as a model reaction to investigate the potential of CO as a molecular trigger for adaptive alkyne hydrogenation (Figure 3a). Catalytic reactions were carried out under batch conditions in Fisher–Porter bottles equipped with magnetic stir bars. Propylene carbonate (PC, 1 mL) was used as a green solvent, the substrate amount was fixed at 500 equivalents relative to the total metal loading, and the reaction proceeded for 16 h at 100°C under either H_2_ (2 bar) or H_2_/CO (P_total_ = 2.5 bar, 4/1 ratio) (see Table S2 and Figure S2 for preliminary tests).

**FIGURE 3:**
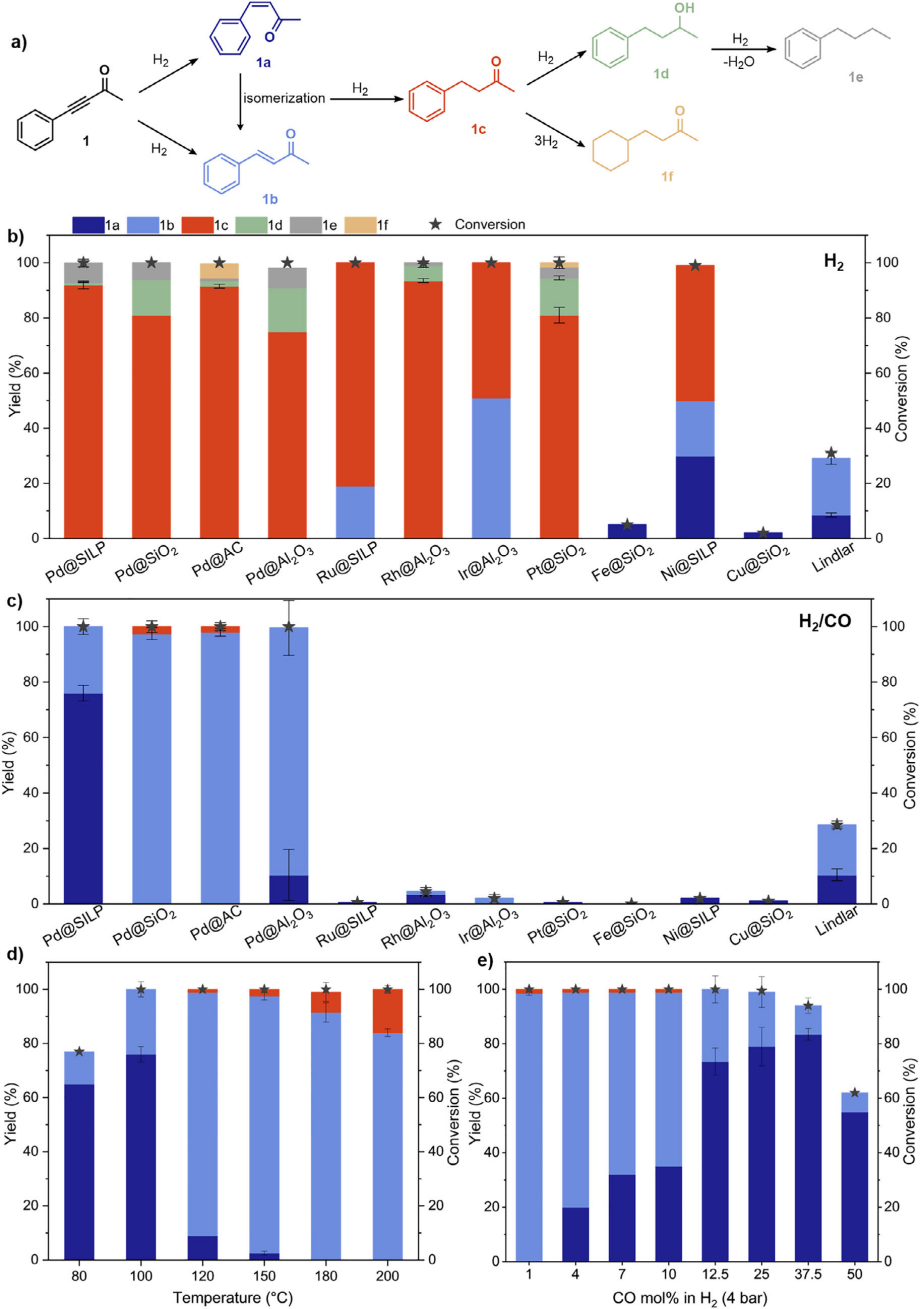
(a) General reaction network for the hydrogenation of **1**. Comparative hydrogenation of **1** catalyzed by different metal NPs catalysts under (b) H_2_ as feed gas and (c) H_2_/CO as feed gas. Hydrogenation of **1** using Pd@SILP under H_2_/CO at (d) different temperatures and (e) different CO concentrations. Reaction conditions: Pd@SILP (10 mg), substrate (1 mmol, 500 eq. w.r.t metal), PC (1 mL), H_2_ (2 bar) or H_2_/CO (P_total_ = 2.5 bar; 4/1) 100°C, 16 h. Yields were determined through GC‐FID using mesitylene as internal standard. Data points are average values of three experiments and error bars represent standard deviations.

With Pd@SILP under H_2_, full hydrogenation of the alkyne functionality occurred to afford 4‐phenylbutan‐2‐one (**1c**) in 91% yield (Figure 3b). This expected typical activity was observed also for reference catalysts composed of Pd NPs immobilized on SiO_2_, activated carbon (AC), and Al_2_O_3_. In sharp contrast, introduction of CO in the H_2_ feed gas led to selective partial hydrogenation of the alkyne to give 4‐phenylbut‐3‐en‐2‐one (**1a**+**1b**) in >99% yield (Figure 3c). Gas‐phase analysis detected no side products from CO hydrogenation (Figure S3). Interestingly, using the Pd@SILP catalyst gave the more valuable *Z* isomer **1a** with high selectivity (74%), while the reference catalysts produced predominantly the thermodynamically favored *E* isomer **1b**. Control experiments subjecting alkene **1a** to the reaction conditions revealed a suppression of the isomerization to **1b** with Pd@SILP as compared to Pd@SiO_2_ indicating that the SILP architecture is pivotal to the observed *Z* stereoselectivity under H_2_/CO (Table S3). This excellent Z stereoselectivity may originate from the confinement of surface Pd sites by CO adsorption and/or interactions with the IL layer, consistent with observations from the literature [47, 48, 49].

While other typical supported metal NPs (Pt, Ru, Rh, Ir, Ni, Fe, Cu; see Table S4) are also capable of hydrogenating the alkyne moiety under H_2_, they all showed a complete loss of activity under H_2_/CO (Figure 3b, c). This behavior is most likely due to a strong poisoning of the metal NPs by CO [50, 51], and highlights the unique capability of Pd NPs to maintain some level of hydrogenation activity in the presence of CO [52, 53]. Notably, Lindlar's catalyst, a benchmark catalyst for alkyne semi‐hydrogenation, gave only 31% yield of **1a**+**1b** under H_2_ with low *Z*‐selectivity (30%) (Figure 3b) and its activity was unchanged under H_2_/CO (Figure 3c). This CO‐insensitivity is attributed to the Pd–Pb alloy, which reduces CO adsorption and surface lifetime by limiting the amount of exposed Pd surface sites [54]. Thus, the CO‐switch gives better control to the alkene products and in case of Pd@SILP provides superior performance toward *Z*‐selective alkyne semi‐hydrogenation as compared to irreversibly Pb‐poisoned Lindlar's catalyst. At the same time, simply using pure H_2_ gives access to alkanes as another valuable product class with the same catalyst in the same reactor under otherwise identical conditions.

The influence of reaction parameters on the CO‐effect was investigated in more detail for the Pd@SILP catalyst. Control experiments in which H_2_ was mixed with Ar, CO_2_, and CH_4_ led to full hydrogenation of the alkyne to product **1c** (Table S5), demonstrating that CO acts as a molecular trigger and not simply as a diluting gas for H_2_. At 80°C incomplete conversion was observed while excellent selectivity toward partial hydrogenation products **1a+1b** was maintained up to 180°C when small amounts of over‐hydrogenated product **1c** started to appear (Figure 3d). Above 120 °C the reaction pathway shifted toward thermodynamic control favoring the formation of *E*‐4‐phenylbut‐3‐en‐2‐one (**1b**) as the major product (90% yield). CO_2_ was detected after reaction at 200°C by gas‐phase analysis, arising from PC decomposition (Figure S4). Excellent selectivity toward semi‐hydrogenation was maintained over a wide CO compositional window (1–50 mol% in H_2_), with a notable dependence of the stereoselectivity on the CO content. The *E* isomer **1b** was predominant up to a CO content of 10 mol%, and the *Z* isomer **1a** became the major product above this value (Figure 3e). This demonstrates the potential that not only the chemo‐, but also the stereo‐selectivity can be adjusted using CO as trigger in hydrogenation over the same catalyst.

Kinetic experiments were conducted to gain deeper insight into the influence of CO on the reaction network over Pd@SILP catalyst (Figure 4). Under pure H_2_, the reaction proceeded to full conversion within less than an hour to product **1b**, followed by its rapid hydrogenation to **1c** (Figure 4a). In contrast, the conversion of **1** was slower under H_2_/CO, and alkene hydrogenation was fully suppressed even after 16 h (Figure 4b). Notably, **1a** was the major product throughout the reaction reaching a maximum selectivity of over 80% at 100% conversion with only slow isomerization to **1b** over time. A ten‐fold increase in substrate/Pd ratio to slow down the reaction failed to reproduce the impact of CO addition (**1b** observed as main intermediate and not **1a**, Figure S5), indicating that CO does not only passivate the catalyst and decrease the overall reaction rate, but also influences the reaction pathway and product selectivity. Replacing H_2_ with D_2_ results in a H/D kinetic isotope effect KIE = 1.5 in the absence of CO. This KIE is consistent with typical values for heterogeneous hydrogenation, and indicates that H_2_ dissociation is unlikely to be rate‐determining [17, 36]. The difference was significantly more pronounced with KIE = 3 for H_2_/CO‐D_2_/CO (Figures 4c, d and S5, S6). As DFT studies have previously showed that CO does not significantly impede dissociative H_2_ adsorption but instead alters Pd ensemble availability and lowers the coverage and mobility of reactive surface hydrogen through competitive adsorption [38, 39], we conclude that the observed primary KIE is most likely associated to the transfer of Pd‐H (or D) to the carbon atom of the substrate. A smaller but significant positive KIE of 1.3 was observed when comparing the initial hydrogenation rates of **1** under H_2_/^12^CO and H_2_/^13^CO (Figure 4e). This is consistent with the known stronger binding of ^13^CO than ^12^CO at metal surfaces [55, 56].

**FIGURE 4:**
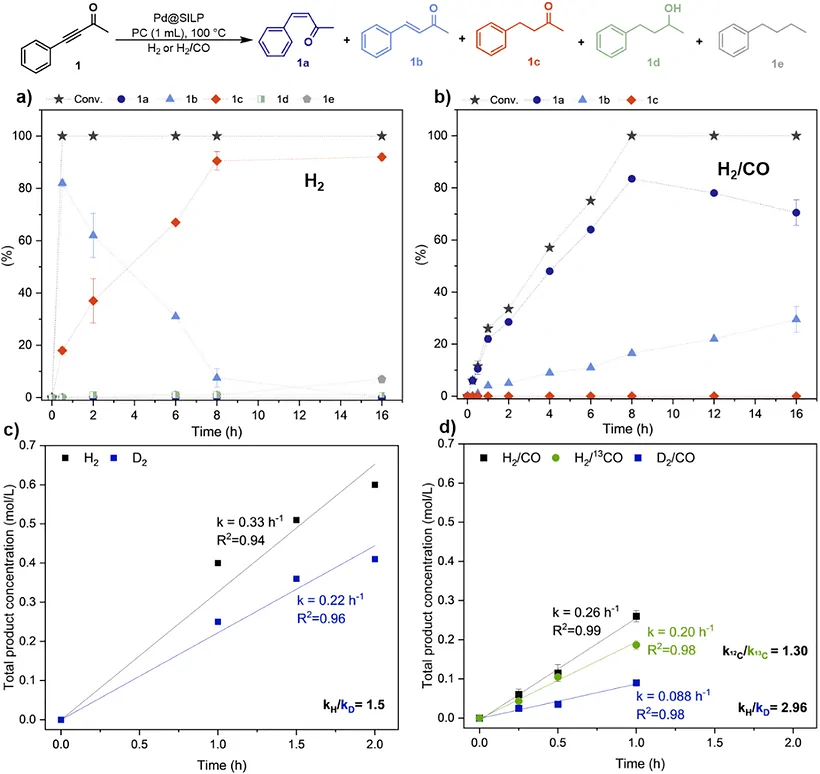
Reaction time profiles for the hydrogenation of **1** under (a) H_2_ and (b) H_2_/CO. Determination of kinetic isotope effects (KIE) for the hydrogenation of **1** under (c) H_2_ and D_2_; (d) H_2_/CO, D_2_/CO, and H_2_/^13^CO. Reaction conditions: Pd@SILP (10 mg, 2 µmol Pd), substrate (1 mmol, 500 eq. w.r.t Pd), PC (1 mL), H_2_ (2 bar) or H_2_/CO (P_total_ = 2.5 bar; 4/1) 100°C (see Supporting Information for detailed procedures). Yields were determined through GC‐FID using mesitylene as internal standard. Data points are average values of three experiments and error bars represent standard deviations.

After the CO‐effect was established, the alternating production of compounds **1c** (90%–95%) and **1a+b** (>99%) in excellent selectivity and yields was validated simply by switching the feed gas composition between H_2_ and H_2_/CO, respectively (Figure 5a). The Pd@SILP catalyst did not require any regeneration or treatment between reaction cycles (see Supporting Information for reversibility test procedure), demonstrating the excellent reversibility and practical simplicity of the selectivity switch over six consecutive cycles (Figures 5a and S7). The Pd@SILP catalyst did not lose activity nor selectivity during the reversibility study or recycling experiments under H_2_ or H_2_/CO (Figure S8). Characterization of the spent catalyst material by ICP‐OES, electron microscopy, and XPS did not show any substantial Pd leaching (Table S1), NPs growth or aggregation (Figures 5b and S9, S10), or changes in electronic properties (Figure S11), respectively. This behavior confirms that the system fully meets the ***R^3^*** rule of adaptive catalytic systems, achieving a truly ***R*** eversible, ***R*** apid, and ***R*** obust control [1].

**FIGURE 5:**
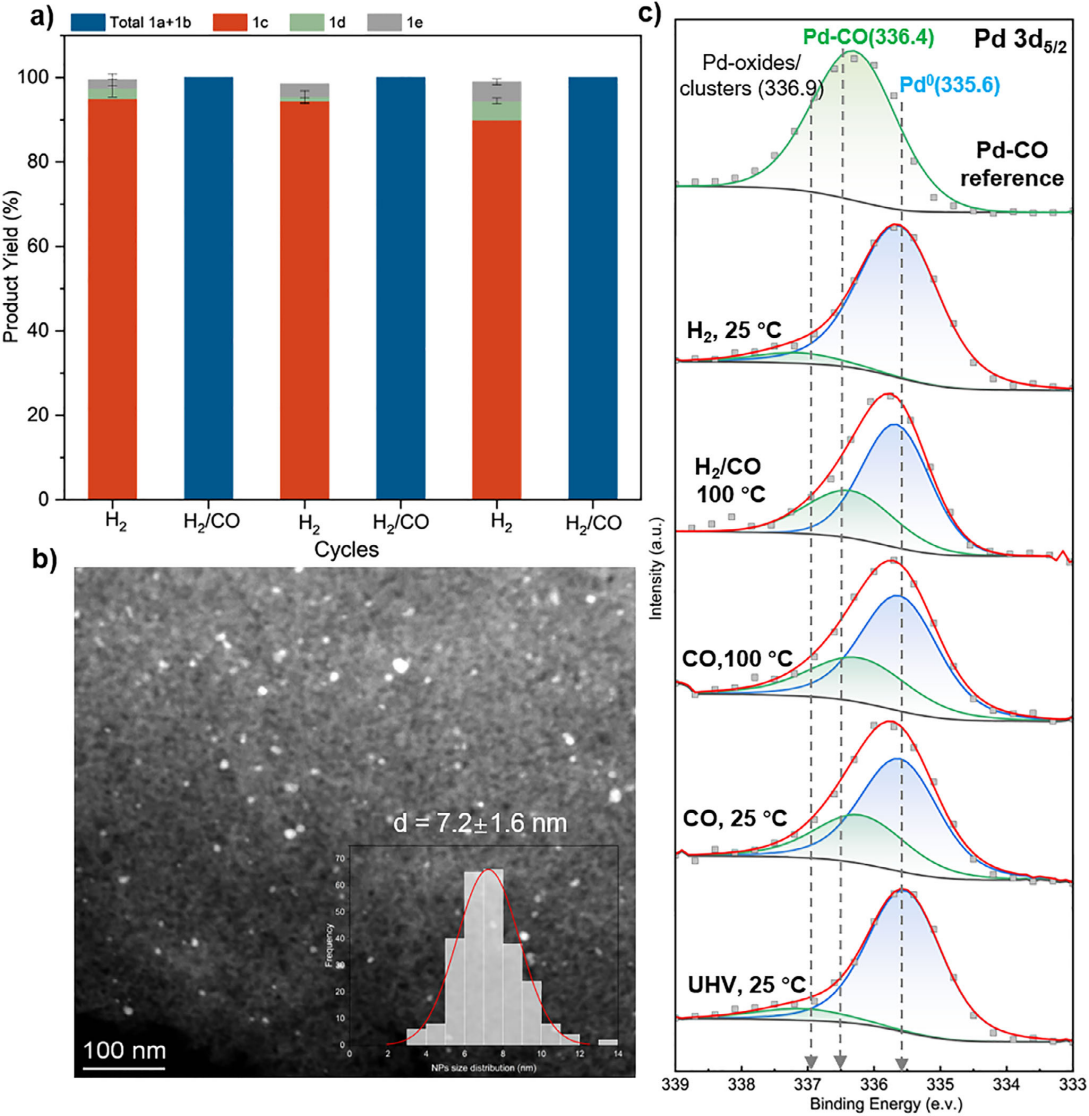
(a) Switchable hydrogenation of **1** over Pd@SILP under alternating H_2_ and H_2_/CO feed gas environment. Reaction conditions: Pd@SILP (50 mg, 10 µmol Pd), **1** (1 mmol, 100 eq. w.r.t Pd), PC (1 mL), H_2_ (2 bar) or H_2_/CO (P_total_ = 2.5 bar; 4/1) 100°C, 2 h. Yields were determined through GC‐FID using mesitylene as internal standard. Data points are average values of three experiments and error bars represent standard deviations. (b) STEM‐HAADF image of Pd after six cycles. (c) In situ NAP‐XPS of a Pd@C reference catalyst under different conditions.

To probe the remarkable CO tolerance and selectivity switch observed with Pd NPs catalysts, in situ NAP‐XPS was performed using Pd@C as a reference material to avoid sample charging with the SILP material (Figure 5c). Under UHV, Pd was found predominantly in the metallic state (Pd3d_5/2_ ∼335.6 eV) with slight surface oxidation (Pd3d_5/2_ ∼336.9 eV). Upon CO exposure at 25°C, the 3d_5/2_ peak maxima shifted toward higher BE and could be deconvoluted into two components corresponding to Pd^0^ and Pd^δ+^─CO (∼336.4 eV), confirming the partial coverage of the Pd surface with CO [57, 58]. The Pd─CO species persisted upon heating the sample to 100°C. Importantly, the spectra remained largely identical under a H_2_/CO (4/1) mixture. However, the Pd─CO contribution rapidly vanished under pure H_2_ at 25°C. These results show a fully reversible process, with adsorption of CO at the Pd surface under H_2_/CO, and its desorption under H_2_. This dynamic displacement of surface CO species as a function of the feed gas composition correlates directly with the selectivity switches observed in Figure 5a.

Collectively, these kinetic and spectroscopic data strongly support competitive adsorption between CO and substrate/intermediate at the Pd NPs surface as the most likely dominant mechanism for the observed selectivity [59].

This is consistent with previously reported DFT studies proposing that surface CO species prevent acetylene over‐hydrogenation through suppression of subsurface hydrogen formation and reduction of the binding energy of alkenes on Pd surfaces, thereby increasing the kinetic barrier for the alkene hydrogenation step [38, 39, 60].

### 2.3 Substrate Scope

The versatility of the CO‐induced adaptivity was explored for a wide substrate scope including internal and terminal alkynes bearing various functionalities. All internal alkynes tested (substrates **1–24**, Table 1) were hydrogenated smoothly to the saturated alkanes under the standard set of reaction conditions (2 bar H_2_, 100°C, 16 h). Gratifyingly, semi‐hydrogenation occurred with high selectivity under H_2_/CO to give the corresponding *Z*‐alkenes in good to excellent yields (products **1a**‐**24a**, 74%–99% yield) under otherwise identical conditions. The utility of both product types accessible from the same substrate by switching only the gas feed with the same catalyst under identical conditions is highlighted for selected examples in the table [61, 62, 63, 64, 65, 66, 67, 68]. The high selectivity for semi‐hydrogenation extended to electron‐donating as well as withdrawing substituents. No dehalogenation occurred for the semi‐hydrogenation (F, Cl, Br in **12–14**), while some C─X cleavage was observed and led to catalyst deactivation under pure H_2_ in case of the bromo‐derivative **14c**. The sulfur‐containing substrates (**20** and **21**) were well tolerated under H_2_/CO, albeit the heterocycle in substrate **21** also reduced the activity under pure H_2_. Notably, functionalities that are typically reduced upon hydrogenation (e.g., CN in **4**, NO_2_ in **5** and **18**, CHO in **19**) were preserved upon use of H_2_/CO (99/1) as the feed gas, outlining the exceptional chemoselectivity of the Pd@SILP catalyst under these conditions. Interestingly, the aliphatic internal alkyne **24** was converted to the *Z*‐alkene **24a** (94%) without any undesired olefin isomerization (Figure S13). Similarly, aromatic and aliphatic terminal alkynes underwent highly selective semi‐hydrogenation under H_2_/CO to yield terminal olefins with no isomerization (**26a**, **27a**) and minimal over‐reduction or oligomerization, while they were fully hydrogenated under pure H_2_ (Table 2). The excellent chemoselectivity under H_2_/CO was illustrated by the retention of functional groups such as NO_2_ (**28**), halogens (**29, 30**) and silyl group (**31**).

**TABLE 1:** Adaptive and chemoselective hydrogenation of internal alkynes using Pd@SILP under H_2_ and H_2_/CO.

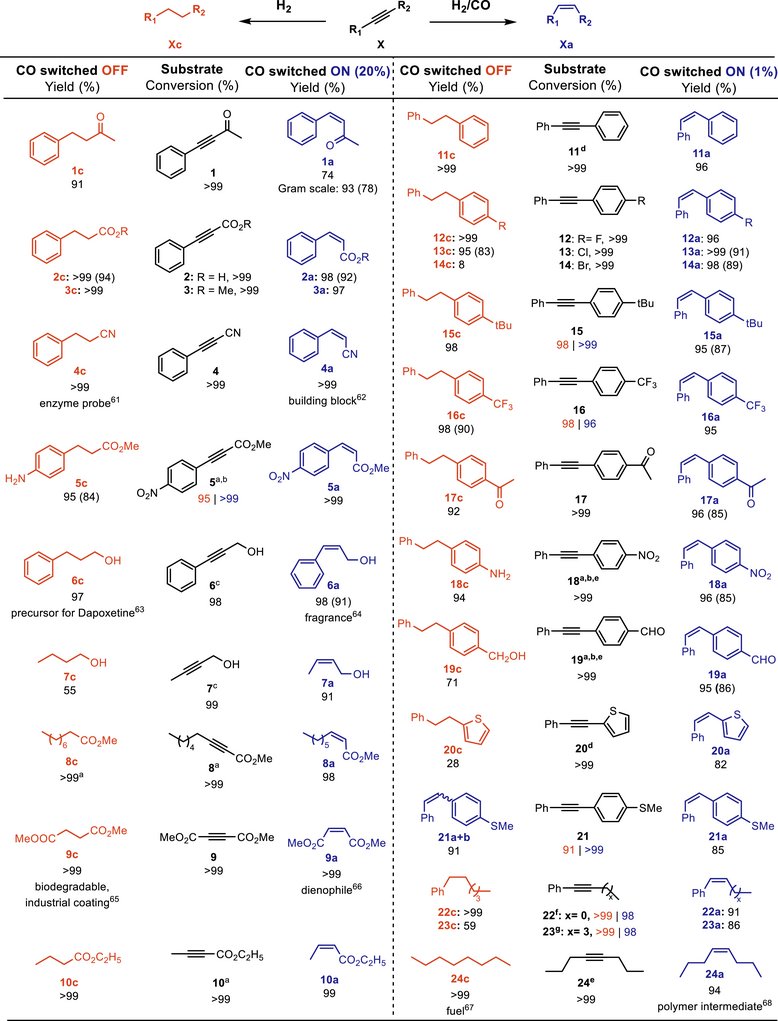

**Reaction conditions**: Pd@SILP (10 mg, 2 µmol Pd), substrate (1 mmol, 500 eq. w.r.t Pd), PC (1 mL), H_2_ (2 bar) or H_2_/CO (left column: P_total_ = 2.5 bar, 4/1, 20% CO and right column: 2 bar, 99/1, 1% CO) 100°C, 16 h. Yields were determined by GC‐FID using mesitylene as internal standard; isolated yields in parentheses. ^a^ H_2_ (4 bar) or H_2_/CO (P_total_ = 4.5 bar; 4/1); ^b^250 eq. substrate; ^c^ solvent = toluene; ^d^ 6 h; ^e^ 16 h; ^f^ 3 h; ^g^ 10 h. Full product distributions are given in Tables S6–S8.

**TABLE 2:** Adaptive and chemoselective hydrogenation of terminal alkynes using Pd@SILP under H_2_ and H_2_/CO.

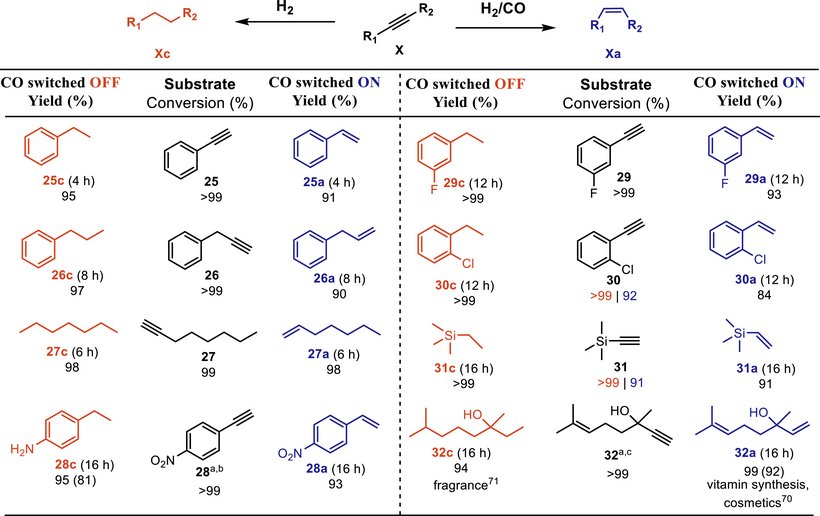

**Reaction conditions**: Pd@SILP (10 mg, 2 µmol Pd), substrate (1 mmol, 500 eq. w.r.t Pd), PC (2 mL), H_2_ (2 bar) or H_2_/CO (P_total_ = 2.5 bar; 4/1) 100°C, time denoted in brackets. Yields were determined through GC‐FID using mesitylene as internal standard; isolated yield in parentheses. ^a^ H_2_ (4 bar) or H_2_/CO (P_total_ = 4.5 bar; 4/1); ^b^ 250 eq. substrate; ^c^ solvent = ethanol.

In industrially relevant applications, the selective removal of trace alkyne impurities from olefin‐rich feedstocks is crucial for high‐performance polymerization processes [69]. As a notable example, the terminal alkyne in dehydrolinalool (**32**) was chemoselectively hydrogenated to the terminal olefin under H_2_/CO while preserving the internal alkene, giving product **32a** in 99% yield which serves as a valuable synthon in vitamin synthesis and cosmetics [70]. In contrast, full reduction of both the triple and double bonds toward the saturated hydrocarbon **32c** (94%, fragrance ingredient) [71] was observed under H_2_.

The broad synthetic utility and robustness of this catalytic system were further validated through the adaptive hydrogenation of structurally complex molecules (**33‐37**), giving selectively two valuable classes of biologically‐relevant products as a function of the feedgas composition (Scheme 1). For instance, ethisterone (**33**) was selectively converted either into the partially reduced steroid derivative **33a** (vinyltestosterone) [72] or into the fully hydrogenated analogue **33c**. Similarly, dehydroisophytol (**34**) underwent selective semi‐hydrogenation under H_2_/CO to furnish the vitamin E precursor [73] **34a** and full hydrogenation to yield dihydroisophytol **34c**, an intermediate in phytane synthesis (lubricant and microbial marker) [74]. This adaptive behavior extends to pharmaceutically relevant scaffolds where **35** was selectively hydrogenated to the resveratrol derivative [26] **35a** or its deeply reduced antiproliferative analogue [75] **35c**. Similarly, propargyl amines **36** and **37** afforded the partially hydrogenated cinnarizine **36a** and naftifine **37a** product [76] as well as the more saturated dopamine transporter inhibitors [77] **36c** and **37c**. In all cases, high chemo‐ and regioselectivity were achieved alongside excellent preservation of sensitive functional groups under H_2_/CO conditions.

**SCHEME 1:**
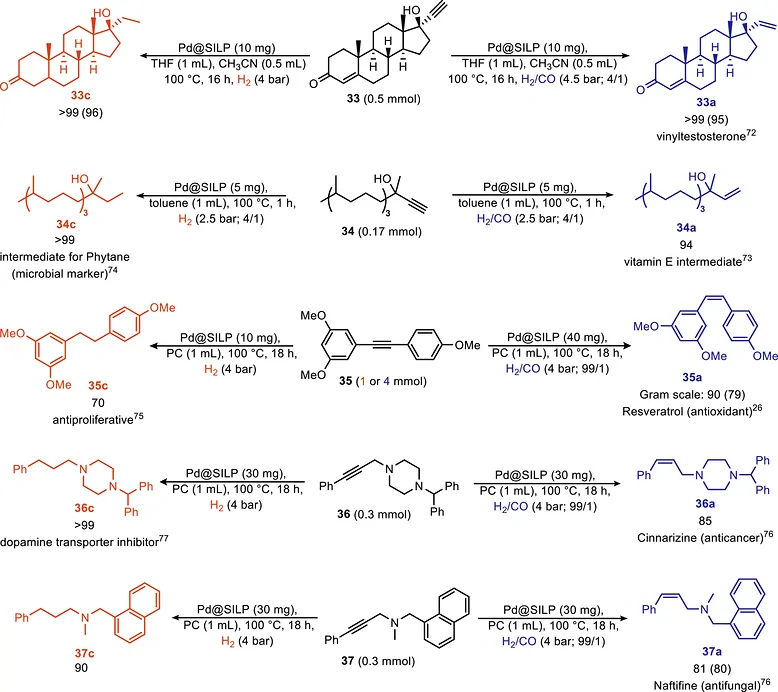
Application of our adaptive catalytic approach to the synthesis of important bioactive molecules. Yields were determined through NMR using mesitylene as internal standard; isolated yields in parentheses.

## 3 Conclusion

In this study, we have successfully demonstrated the potential of CO as a molecular trigger to enable adaptive catalysis. As a synthetically‐relevant case study, the reversible, rapid, and robust selectivity switch between alkane and *Z*‐alkene products in alkyne hydrogenation has been achieved using Pd@SILP under H_2_ or H_2_/CO, respectively. Detailed mechanistic investigations suggest that the dynamic adsorption of CO competing with alkene intermediates at the Pd surface is responsible for the observed selectivity switch. The outstanding versatility and functional‐group tolerance of the approach were demonstrated via the adaptive hydrogenation of 37 structurally diverse alkyne‐containing substrates, giving valuable alkane (55%–99% yield) and *Z*‐alkene (74%–99% yield) products, many of which find important applications in the pharmaceutical industry. In the presence of CO, *E*‐alkenes are also accessible by variations of reaction conditions or with other supported Pd catalysts. The results reported here have significant implications for the development of sustainable chemical manufacturing and the creation of high‐value intermediates for pharmaceuticals, agrochemicals, and fine chemicals. In particular, the excellent functional‐group tolerance of the present system eliminates the need for protection–deprotection strategies typically required to prevent over‐reduction or loss of selectivity in functionally dense molecules. Thus, this approach can streamline synthetic routes and enhance step economy in the synthesis of complex and biologically relevant compounds. In contrast to its notorious reputation as a catalyst poison, the use of CO as a molecular trigger defines a new paradigm for its role in catalytic hydrogenation through the design of adaptive catalytic systems, with potential applications going far beyond the demonstrated on‐demand selectivity switching in alkyne hydrogenation.

## Author Contributions

**Manisha Durai**: investigation, writing – original draft, writing – review and editing, data curation, methodology, visualization, formal analysis. **Lachlan Sharp‐bucknall**: writing – review and editing, investigation, data curation, methodology. **Tim Alexander Schubert**: investigation, data curation. **Jacob Johny**: investigation, methodology, data curation, writing – review and editing. **John‐Tommes Krzeslack**: investigation, data curation, methodology. **Walid Hetaba**: investigation, writing – review and editing, validation, methodology. **Walter Leitner**: conceptualization, funding acquisition, writing – review and editing, validation, project administration, resources, supervision. **Alexis Bordet**: conceptualization, writing – review and editing, validation, project administration, resources, supervision, data curation, investigation, formal analysis.

## Conflicts of Interest

The authors declare no conflicts of interest.

## Supporting information




**Supporting File**: The Supporting Information containing detailed experimental procedure, supplementary and analytical data is available free of charge.

## Data Availability

The data that supports the findings of this study are available in the supplementary material of this article. In addition, source data will be available for download on Edmond (open data repository of the Max Planck Society) at https://doi.org/10.17617/3.H69N6E.
