# Supplementary material for: Adaptive Hydrogenation of Alkynes Using CO as a Molecular Trigger to Selectively Produce Alkanes or Z‐Alkenes
Source: Angew Chem Int Ed Engl. 2026 Aug 6;65(44):e9302519. doi: 10.1002/anie.9302519 (PMC13703942; doi:10.1002/anie.9302519)
Supplement: Supplementary file 1 [file ANIE-65-e9302519-s001.docx]

**Supporting Information**

**Adaptive Hydrogenation of Alkynes Using CO as a Molecular Trigger to Selectively Produce Alkanes or *Z*-alkenes**

*Manisha Durai,^a,b^ Lachlan Sharp-Bucknall,^a^ Tim Alexander Schubert,^a^ Jacob Johny,^a^* *John-Tommes Krzeslack*, *^a^ Walid Hetaba,^a^ Walter Leitner^*a,b^ and Alexis Bordet^*a^*

[a]	Max Planck Institute for Chemical Energy Conversion, Stiftstr. 34-36, 45470 Mülheim an der Ruhr,

Germany. E-Mail: [alexis.bordet@cec.mpg.de](mailto:alexis.bordet@cec.mpg.de), [walter.leitner@cec.mpg.de](mailto:walter.leitner@cec.mpg.de)

[b]	Institut für Technische und Makromolekulare Chemie, RWTH Aachen University, Worringerweg 2, 52074 Aachen, Germany

Contents

[1. General and Safety Warning	2](#_Toc235094715)

[3. Analytics	2](#_Toc235094716)

[4. Synthesis of Pd@SILP	4](#_Toc235094717)

[5. Synthesis of Ru@SILP	6](#_Toc235094718)

[6. Synthesis of Fe@SiO_2_	6](#_Toc235094719)

[7. Synthesis of Ni@SILP	6](#_Toc235094720)

[8. Synthesis of Cu@SiO_2_	6](#_Toc235094721)

[9. Synthesis of Pd@SiO_2_	7](#_Toc235094722)

[10. General Procedure for the synthesis of alkyne substrates	7](#_Toc235094723)

[11. General procedure for adaptive hydrogenation of alkynes	8](#_Toc235094724)

[12. Gram-scale Synthesis	8](#_Toc235094725)

[13. Catalyst Recycling	9](#_Toc235094726)

[14. Reversibility experiment	9](#_Toc235094727)

[15. Supplementary Tables, Figures, and Schemes	10](#_Toc235094728)

[6. Analytical data for substrates	22](#_Toc235094729)

[17. Analytical data for Isolated products	28](#_Toc235094730)

[18. ^1^H and ^13^C NMR spectra of isolated substrates and products	36](#_Toc235094731)

[19. References	70](#_Toc235094732)

# 1. General and Safety Warning

All experiments with compressed CO or H_2_ were carried out in a Fisher-Porter bottles placed behind a protective blast shield. The catalytic tests were repeated at least two times to ensure reproducibility. The synthesis of ionic liquids (ILs), Pd@SILP, and reference catalysts were carried out under an argon atmosphere using standard Schlenk techniques or in a glovebox, as previously reported or with modified protocols. Solvents were used directly without purification or drying. Silica from Merck (Grade 10184, pore size 100 Å, 63–200 µm) was dehydroxylated under vacuum at 500 °C for 16 h. Pd_2_(dba)_3_ was obtained from Sigma Aldrich. Substrates 1-4, 6-11, 14, 22-34 were obtained from commercial sources like Sigma Aldrich, BLD pharm, TCI chemicals and Thermo Fisher Scientific. All catalytic experiments were performed under air.

# 3. Analytics

**Solution-state NMR**

All solution-state NMR spectra were recorded on a Bruker Ascend 400 MHz and 500 MHz spectrometer at room temperature. The coupling constants (*J*) are given in Hertz (Hz), and the chemical shifts (*δ*) are expressed in ppm, relative to TMS at 25 °C. The peak multiplicities were designated as follows: s = singlet; d = doublet; t = triplet, dd = doublet of doublet, ddd = doublet of doublets of doublets, dt = doublet of triplet, m = multiplet.

**Gas Chromatography**

Gas chromatography (GC) was performed on a Shimadzu GC-2030 equipped with an FID-detector and a Rtx-1701 column from Restek.

**Scanning Transmission Electron Microscopy with Energy Dispersive X- ray Spectroscopy**

STEM/EDX was performed on a Thermo Scientific Talos F200X operated at 200 kV acceleration voltage. STEM imaging was performed using a camera length of 98 mm and a beam current of 99 pA (measured on the screen). EDX data was acquired using the SuperX EDX system, incorporating four SDDs with a total of 0.9sr collection angle. Samples for electron microscopy were prepared by depositing the colloidal powder onto a copper TEM grid with an amorphous carbon support film. All samples were handled under inert conditions from preparation to insertion into the TEM. To determine the NP size, the particles were analyzed using ImageJ with a count of at least 150–200 NPs.

**N_2_ Adsorption and Brunauer-Emmett-Teller Surface Area Determination**

N_2_ adsorption measurements were performed on a Quadrasorb SI from Quantachrom Instruments. BET surface areas were determined using a QuadraSorb station (7.01).

**X-ray Photoelectron Spectroscopy**

XPS measurements were performed on fresh and recycled Pd@SILP where the corresponding powder samples were spread onto a carbon tape on the sample holder in a compact manner. For the measurements, a near ambient pressure (NAP) XPS (SPECS GmbH) at ultra-high vacuum conditions (~10^-8^ mbar) was employed. The system was equipped with an Al-Kα source which produces monochromated X-rays of energy 1486.6 eV, and a NAP hemispherical energy analyzer with an inbuilt double delay line detector. All high-resolution spectra were recorded using a pass energy of 20 eV and a resolution of 0.05 eV whereas the survey scans were recorded using 100 eV pass energy at a resolution of 0.1 eV. In each case, at least 10 consecutive scans were performed. Data analysis was performed using the CasaXPS software after a binding energy calibration using the C1s at 484.6 eV. Near-ambient pressure X-ray photoelectron spectroscopy (NAP-XPS) measurements were performed with Pd@C sample using a PHOIBOS 150 R3 NAP HR hemispherical analyzer equipped with a 1D-DLD detector. X-ray excitation was provided by a monochromated XR50MF Al Kα source (hν = 1486.6 eV). The X-ray spot size was ~1 mm, and the analyzer nozzle diameter was 800 µm. Heating ramps were applied at 1 K s⁻¹ from lower to higher temperatures. Survey spectra were acquired with a pass energy of 100 eV and a step size of 1.0 eV. High-resolution spectra were recorded with a pass energy of 20 eV and a step size of 0.1 eV. The sample was heated with given gas atmosphere for ~20 mins before cooling or switching the environment.

**Inductively Coupled Plasma Optical Emission Spectroscopy (ICP-OES)**

ICP was carried out by Mikroanalytisches Labor Kolbe on an ICP-OES Spectro Arcos from Spectro. The sample preparation was performed using a CEM-Mars 6 microwave.

# 4. Synthesis of Pd@SILP

4.1. Synthesis of ionic liquid

Ionic liquid and SILP were synthesized using literature procedure.^[1]^

*-Synthesis of IL-[1-1-butyl-triethoxysilylpropyl-imidazolium]Br*

1-bromobutane (1.28 g, 9.34 mmol, 1.2 eq.) was weighed in a Schlenk flask in the glovebox and dried under vacuum prior to use, then dissolved in 40 mL anhydrous toluene. N-(3-triethoxysilylpropyl) imidazole (2.1 g, 7.7 mmol, 1.00 eq.) was weighed separately into a Schlenk flask in the glovebox and added to the 1-bromobutane solution. The mixture was refluxed for 18 h at 130 °C under Ar. After cooling down the reaction, the obtained mixture was washed 3 times with 10 mL pentane then dried in vacuum at RT for 15 h to form a viscous orange oil (73 % yield).

**^1^H NMR (400 MHz, (CD_3_)_2_CO,** δ**):** 10.45 (s, 1H), 8.01 (s, 1H), 7.96 (s, 1H), 4.48 (td, *J* = 7.2, 4.4 Hz, 4H), 3.80 (q, *J* = 7.0 Hz, 6H), 1.95 (m, 2H), 1.38 (m, 2H), 1.17 (t, *J* = 7.0 Hz, 9H), 0.94 (t, *J* = 7.4 Hz, 3H), 0.64 (m, 2H).

**^13^C NMR (100.6 MHz, (CD_3_)_2_CO,** δ**):** 137.75 (s, 1C), 122.97 (s, 1C), 122.99 (s, 1C), 58.71 (s, 3C), 52.10 (s, 1C), 49.45 (s, 1C), 32.57 (s, 1C), 24.81 (s, 1C), 19.75 (s, 1C), 18.42 (s, 3C), 13.50 (s, 1C), 7.49 (s, 1C).

*-Synthesis of IL-[1-1-butyl-triethoxysilylpropyl-imidazolium]NTf_2_*

For the anion exchange, no Schlenk techniques were used. 1-butyl-3,3-triethoxysilylpropyl imidazolium bromide (1.13 g, 2.8 mmol, 1 eq.) was dissolved in 20 mL DCM. In a separate ﬂask, Lithium bis(trifluoromethane)sulfonimide (LiNTf_2_) (0.9 g, 3.1 mmol, 1.05 eq.) was dissolved in 10 mL water. The solutions were mixed and vigorously stirred for 30 min at room temperature. The organic phase was washed three times with water, dried over MgSO_4_ and the solvent was removed under reduced pressure. The resulting viscous liquid was dried overnight at 60 °C in vacuo. After drying, a thick yellow/brown oil was obtained (66% yield).

**^1^H NMR (400 MHz, (CD_3_)_2_CO,** δ**):** 9.13 (s, 1H), 7.83 (m, 2H), 4.40 (m, 4H), 3.80 (q, *J* = 7.0 Hz, 6H), 1.95 (m, 2H), 1.38 (m, *J* = 14.8, 7.4 Hz, 2H), 1.18 (t, *J* = 7.0 Hz, 9H), 0.95 (t, *J* = 7.4 Hz, 3H), 0.62 (m, 2H).

**^13^C NMR (100.6 MHz, (CD_3_)_2_CO,** δ**):** 136.47 (s, 1C), 123.38 (s, 1C), 123.32 (s, 1C), 58.69 (s, 3C), 52.44 (s, 1C), 50.07 (s, 1C), 32.39 (s, 1C), 24.63 (s, 1C), 19.72 (s, 1C), 18.35 (s, 3C), 13.36 (s, 1C), 7.48 (s, 1C).

**^19^F NMR (376 MHz, (CD_3_)_2_CO,** δ**):** - 79.91.

4.2. Synthesis of SILP

[1-1-butyl-triethoxysilylpropyl-imidazolium]NTf_2_ (5.49 g, 8.80 mmol) was dissolved in toluene (20 mL) and added to a suspension of partially dehydroxylated SiO_2_ (10.0 g in 50.0 mL toluene). The resulting mixture was refluxed for 18 h. Upon removal of the organic phase, the SILP was washed with DCM (3x25 mL) and dried in vacuo. The organic phases were combined and solvent removed to determine the residual quantity of IL not grafted onto the dehydroxylated SiO_2_ (Total IL Loading = Theoretical Loading – Recovered Residual IL). Ionic liquid loading was found to be 0.67 mmol/g.

4.3. Synthesis of Pd@SILP

SILP (1 g) was taken in a Fisher-Porter bottle (70 mL) along with mesitylene (1 mL). Pd_2_(dba)_3_ (92 mg, 0.1 mmol) was dissolved in mesitylene (2 mL) and added to the bottle. the suspension was stirred under argon at r.t. for 1 h. The Fisher-Porter bottle was evacuated and backfilled with H_2_. The suspension was stirred under H_2_ (3 bar) at 150 °C for 18 h. The supernatant was removed and Pd@SILP (2 wt% Pd) was washed with toluene (3 x 5 mL) and dried in vacuo at 45 °C for 1 h.

# 5. Synthesis of Ru@SILP

[Ru(2-methylallyl)_2_(cod)] (64 mg, 0.2 mmol) in THF (5 mL) was added to a suspension of partially dehydroxylated (500 °C under vacuum for 16 h) SiO_2_ (1.0 g) in THF (10 mL) and stirred for 1 h at room temperature.^[1]^ After removal of solvent under vacuum, the impregnated SiO_2_ was transferred to a 10 mL high-pressure autoclave containing a glass inlet and heated at 100 °C under an atmosphere of H_2_ (50 bar) for 18 h. The autoclave was then cooled and Ru@SiO_2_ was obtained as a black powder.

# 6. Synthesis of Fe@SiO_2_

Inside a glovebox, PA (2.6 mmol, 1332.8 mg) and HDA (2 mmol, 966 mg) were added to a green solution of (Fe[N(SiMe_3_)_2_]_2_)_2_ (0.5 mmol, 1506 mg) in distilled and degassed mesitylene (80 mL) in a Fisher Porter bottle. The bottle was pressurized with H_2_ (4 bar) and heated in an oil bath at 150 °C for 48 h under vigorous magnetic stirring. After cooling to room temperature, the reaction was stopped, and the NPs were recovered by magnetic decantation and washed three times with toluene (3 × 10 mL) and three times with THF (3 × 10 mL), followed by drying under vacuum. 10 mg of the resulting Fe nanoparticles^[2]^ were taken in a flask with 250 mg of SiO_2_ in THF (4 mL) and stirred overnight at rt. The solvent was removed and dried under vacuum.

# 7. Synthesis of Ni@SILP

SILP (500 mg) was taken in a Fisher-Porter bottle (70 mL) along with mesitylene (1 mL). Ni(COD)_2_ (117 mg, 0.4 mmol) was dissolved in mesitylene (2 mL) and added to the bottle.^[3]^ the suspension was stirred under argon at r.t. for 1 h. The Fisher-Porter bottle was evacuated and backfilled with H_2_. The suspension was stirred under H_2_ (3 bar) at 150 °C for 18 h. The supernatant was removed and Ni@SILP was washed with toluene (3 x 5 mL) and dried in vacuo at 45 °C for 1 h.

# 8. Synthesis of Cu@SiO_2_

SiO_2_ (250 mg) was taken in a Fisher-Porter bottle (70 mL) along with mesitylene (1 mL). Mesityl copper (183 mg, 0.2 mmol) was dissolved in mesitylene (2 mL) and added to the bottle.^[4]^ the suspension was stirred under argon at r.t. for 1 h. The Fisher-Porter bottle was evacuated and backfilled with H_2_. The suspension was stirred under H_2_ (3 bar) at 150 °C for 18 h. The supernatant was removed and Cu@SiO_2_ was washed with toluene (3 x 5 mL) and dried in vacuo at 45 °C for 1 h.

# 9. Synthesis of Pd@SiO_2_

Similar to Pd@SILP synthesis, SiO_2_ (500 mg) was taken in a Fisher-Porter bottle (70 mL) along with mesitylene (1 mL). Pd_2_(dba)_3_ (46 mg, 0.05 mmol) was dissolved in mesitylene (2 mL) and added to the bottle. the suspension was stirred under argon at r.t. for 1 h. The Fisher-Porter bottle was evacuated and backfilled with H_2_. The suspension was stirred under H_2_ (3 bar) at 150 °C for 18 h. The supernatant was removed and Pd@ SiO_2_ was washed with toluene (3 x 5 mL) and dried in vacuo at 45 °C for 1 h.

# 10. General Procedure for the synthesis of alkyne substrates

In a typical synthesis, an oven-dried schlenk flask was charged with CuI (0.2 mmol, 0.04 equiv.), Pd(PPh_3_)_2_Cl_2_ (0.1 mmol, 0.04 equiv.), aryl halide (5 mmol, 1 equiv.), phenylacetylene (6 mmol, 1.2 equiv.), Et_3_N (15 mmol) and THF(15 mL) under argon. The mixture was stirred at rt (or at 80 °C for aryl bromides) for 16 h. Upon completion, the reaction mixture was brought to room temperature and filtered over celite; the pad being washed with EtOAc (2 × 10 mL). Volatiles were then removed in vacuo where the crude residue was purified by silica gel column chromatography (Pentane:EtOAc)to afford alkyne substrates 12, 13, 15-21 and 36.

Substrate 5 was synthesized using literature procedure.^[5]^ A sealed flask was charged with 1-ethynyl-4-methoxybenzene (3.75 mmol, 1.0 equiv) in THF (7 mL) under argon and cooled to ₋78 °C. n-Butyllithium (2.5 M, 7.9 mmol, 1.05 equiv) was added dropwise and stirred for 30 min. Iodomethane (4.2 mmol, 1.1 equiv) was then added, and the mixture was warmed to room temperature and stirred for 16 h. The reaction was recooled to ₋78 °C, quenched with 2-propanol and water, and extracted with CH_2_Cl_2_. The combined organic layers were washed with brine, dried over Na_2_SO_4_, and concentrated. The crude product was purified by silica gel chromatography.

Substrates 37 and 38 were synthesized through A3 coupling as follows.^[6]^ In a typical experiment, Cu(I)Cl (40 mg, 0.4 mmol Cu), paraformaldehyde (5 mmol), alkyne (5.2 mmol), and amine (5.2 mmol) were loaded in a 50 mL Schlenk tube. Toluene (5 mL) was added and the resulting mixture was placed in an oil bath preheated at 70 °C for 12 h. Volatiles were then removed in vacuo where the crude residue was purified by silica gel column chromatography (Pentane:EtOAc) affording the corresponding propargylic amine susbtrates.

# 11. General procedure for adaptive hydrogenation of alkynes

*11.1. Hydrogenation of alkynes using H_2_/CO as feed gas:*

Typically, Pd@SILP (10 mg, 0.002 mmol Pd), alkyne (1 mmol, 500 equiv. w.r.t. Pd), propylene carbonate (1 mL) were added to a Fisher-Porter (FP) bottle with a stirring bar. The reaction vessel was sealed, evacuated and pressurized with 2 bar H_2_/0.5 bar CO for substrates **1-10, 25-32, 35-36** and 2 bar H_2_ containing 1% CO for substrates **11-24**, **37-39**. The FP bottle was then placed behind a protective blast shield and heated at 100 $^{\circ}$C for 6-16 h. After reaction completion, the bottle was cooled down and excess pressure was released carefully. Conversion and yield were determined by GC-FID using mesitylene as internal standard.

*11.2. Hydrogenation of alkynes using H_2_ as feed gas:*

Typically, Pd@SILP (10 mg, 0.002 mmol Pd), alkyne (1 mmol, 500 equiv. w.r.t. Pd), propylene carbonate (1 mL) were added to a Fisher-Porter (FP) bottle with a stirring bar. The reaction vessel was sealed, evacuated and pressurized with 2 bar H_2_. The FP bottle was then placed behind a protective blast shield and heated at 100 $^{\circ}$C for 16 h. After reaction completion, the bottle was cooled down and excess pressure was released carefully. Conversion and yield were determined by GC-FID using mesitylene as internal standard.

# 12. Gram-scale Synthesis

Pd@SILP (80 mg), **1** (8 mmol, 500 equiv. w.r.t. Pd), propylene carbonate (3 mL) were added to a Fisher-Porter (FP) bottle with a stirring bar. The reaction vessel was sealed, evacuated and pressurized with 4 bar H_2_ and 0.5 bar CO. The FP bottle was then placed behind a protective blast shield and heated at 100 $^{\circ}$C for 5 h. After reaction completion, the bottle was cooled down and excess pressure was released carefully. Conversion and yield were determined by GC-FID using mesitylene as internal standard. The reaction mixture was separated by filtration, rinsed with ethyl acetate (EtOAc) and washed with brine. The organic layer was collected, dried over MgSO_4_ and concentrated by rotary evaporation. The resulting crude product was purified by flash column chromatography on silica gel using EtOAc/pentane as eluent to obtain pure target compound (Z isomer) in 78% isolated yield (911 mg)**.**

# 13. Catalyst Recycling

13.1 Recycling under H_2_/CO

Pd@SILP (20 mg, 0.004 mmol Pd), alkyne (1 mmol, 250 equiv. w.r.t. Pd), propylene carbonate (1 mL) were added to a Fisher-Porter (FP) bottle with a stirring bar. The reaction vessel was sealed, evacuated and pressurized with 2 bar H_2_ and 0.5 bar CO. The FP bottle was then placed behind a protective blast shield and heated at 100 $^{\circ}$C for 2 h. After reaction completion, the bottle was cooled down and excess pressure was released carefully. The supernatant was removed to prepare a GC sample and the solid catalyst was washed once with propylene carbonate (1.5 mL). New substrates and solvent were added to the washed catalyst to proceed the next cycle.

13.2 Recycling under H_2_

Pd@SILP (20 mg, 0.004 mmol Pd), alkyne (1 mmol, 250 equiv. w.r.t. Pd), propylene carbonate (1 mL) were added to a Fisher-Porter (FP) bottle with a stirring bar. The reaction vessel was sealed, evacuated and pressurized with 2 bar H_2_. The FP bottle was then placed behind a protective blast shield and heated at 100 $^{\circ}$C for 2 h. After reaction completion, the bottle was cooled down and excess pressure was released carefully. The supernatant was removed to prepare a GC sample and the solid catalyst was washed once with propylene carbonate (1.5 mL). New substrates and solvent were added to the washed catalyst to proceed the next cycle.

# 14. Reversibility experiment

Pd@SILP (50 mg, 0.01 mmol Pd), alkyne (1 mmol, 100 equiv. w.r.t. Pd), propylene carbonate (1 mL) were added to a Fisher-Porter (FP) bottle with a stirring bar. The reaction vessel was sealed, evacuated and pressurized with desired gas. The FP bottle was then placed behind a protective blast shield and heated at 100 $^{\circ}$C for 2 h. After reaction completion, the bottle was cooled down and excess pressure was released carefully. The supernatant was removed to prepare a GC sample and the solid catalyst was washed once with propylene carbonate (1.5 mL). New substrates and solvent were added to the washed catalyst to proceed the next cycle. This process was repeated for each cycle by alternatively changing the feed gas to H_2_ and H_2_/CO.

# 15. Supplementary Tables, Figures, and Schemes

**Table S1.** Characterization of different materials by N_2_ physisorption and ICP-OES.

| Entry | Material | BET surface area (m^2^.g^-1^) | Pd loading (ICP-OES) (wt%) |
| --- | --- | --- | --- |
| 1 | SiO_2_ | 332 | - |
| 2 | SILP | 239 | - |
| 3 | Pd@SILP | 262 | 1.99 |
| 4 | After catalysis under H_2_ | 298 | 1.98 |
| 5 | After catalysis under H_2_/CO | 259 | 1.96 |

**Figure S1**. Size distribution of Pd nanoparticles in Pd@SILP. Average diameter = 7.2±2.0 nm.

**Table S2.** Variation of reaction conditions in the hydrogenation of **1** using Pd@SILP.

| Entry | Cat.  loading  (µmol Pd) | P_H2_ (bar) | P_CO_ (bar) | Time (h) | Conv. (%) | Yield **1a+1b** (%) | Yield **1c** (%) | Yield **1e** (%) |
| --- | --- | --- | --- | --- | --- | --- | --- | --- |
| 1 | 10 | 2 | - | 2 | >99 | - | 95 | 4 |
| 2 |  | 2 | 0.5 |  | >99 | >99(Z:E=73:27) | - | - |
| 3 | 4 | 2 | - | 2 | >99 | - | 93 | 6 |
| 4 |  | 2 | 0.5 |  | 79 | 79(Z:E= 78:22) | - | - |
| 5 | 2 | 2 | - | 2 | >99 | - | 94 | 5 |
| 6 |  | 2 | 0.5 |  | 33 | 33(Z:E = 84:16) | - | - |
| 7 | 2 | 2 | - | 16 | >99 | - | 91 | 8 |
| 8 |  | 2 | 0.5 |  | >99 | >99(Z:E= 4:26) | - | - |

Reaction conditions: **1** (1 mmol), Pd@SILP (w.r.t **1**), PC (1 mL), H_2_ (2 bar) or H_2_/CO (P_total_ = 2.5 bar; 4/1) 100 °C. Conversion and yield were determined through GC-FID using mesitylene as internal standard denoted as product yield (Z:E).

**Figure S2.** Optimization of solvent for the semi-hydrogenation of **1** to **1a and 1b**. Reaction Conditions: Pd@SILP (10 mg), **1** (1 mmol, 500 eq. w.r.t Pd), solvent (1 mL), H_2_/CO (P_total_ = 2.5 bar; 4/1) 100 °C, 2h. Conversion and yield were determined through GC-FID using mesitylene as internal standard.

**Figure S3.** Headspace (a) GC-TCD and (b) GC-FID of semihydrogenation of **1.** Reaction conditions: **1** (1 mmol, 500 eq. w.r.t Pd), Pd@SILP (10 mg), PC (1 mL), H_2_/CO (P_total_ =2.5 bar; 4/1) 100 °C, 16 h.

**Table S3.** Reference experiments: *Z* to *E* isomerization using different catalysts.

| Entry | Catalyst | Conversion (%) | Yield **1b**(%) |
| --- | --- | --- | --- |
| 1 | Pd@SILP | 6 | 6 |
| 2 | Pd@SiO_2_ | 89 | 89 |
| 3 | SILP | 10 | 10 |
| 4 | SiO_2_ | 11 | 11 |

**Table S4.** Details of the reference catalysts used in this study.

| Catalyst | Source | Metal Loading (wt%) | NPs Size (nm) |
| --- | --- | --- | --- |
| Ru@SILP | synthesized | 2 | 1.8±0.4 |
| Rh@Al_2_O_3_ | Abcr GmbH | 5 | 2.1±0.5 |
| Ir@Al_2_O_3_ | Thermo Fisher scientific | 1 | 3.5±1.6 |
| Pt@SiO_2_ | Sigma Aldrich | 5 | 2.4±0.9 |
| Fe@SiO_2_ | synthesized | 4 | 10.1±1.8 |
| Ni@SILP | synthesized | 5 | 10.5±2.7 |
| Cu@SiO_2_ | synthesized | 5 | 3.5±0.8 |
| Pd@SiO_2_ | synthesized | 2 | 4.1±1.0 |
| Pd@AC | Sigma Aldrich | 5 | 2.4±0.7 |
| Pd@Al_2_O_3_ | Sigma Aldrich | 5 | 3.2±0.9 |

**Figure S4.** Headspace GC-TCD of a) semihydrogenation of **1** at 200 °C using H_2_/CO and b) reference blank experiment using Pd@SILP (10 mg), PC (1 mL), 200 °C.

**Table S5.** Reference experiments using different gas for hydrogenation of **1** using Pd@SILP.

| Entry | P_H2_ (bar) | Px (bar) | Conv. (%) | Yield **1a+1b** (%) | Yield **1c** (%) | Yield **1d** (%) | Yield **1e** (%) |
| --- | --- | --- | --- | --- | --- | --- | --- |
| 1 | 2 | - | >99 | - | 93 | - | 7 |
| 2 | 2 | CO (0.5) | >99 | >99 (74:26) | - | - | - |
| 3 | 2 | Ar (0.5) | >99 | 1 (0:100) | 93 | - | 6 |
| 4 | 2 | CO_2_ (0.5) | >99 | 2 (0:100) | 90 | - | 7 |
| 5 | 2 | CH_4_ (0.5) | >99 | - | 90 | 2 | 7 |

Reaction Condition: Pd@SILP (10 mg), **1** (1 mmol, 500 eq. w.r.t Pd), PC (1 mL), H_2_ (2 bar), H_2_/CO (P_total_ = 2.5 bar; 4/1) 100 °C, 16 h. Conversion and yield were determined through GC-FID using mesitylene as internal standard.

**Figure S5.** Time profiles for hydrogenation of **1** under H_2_ and D_2_. Reaction conditions: Pd@SILP (10 mg, 2 µmol Pd), **1** (10 mmol, 5000 eq. w.r.t Pd), PC (1 mL), H_2_ or D_2_ (2 bar) 100 °C. Yields were determined through GC-FID using mesitylene as internal standard.

**Figure S6.** Time profiles for hydrogenation of **1** under D_2_/CO. Reaction conditions: Pd@SILP (10 mg), **1** (1 mmol, 500 eq. w.r.t Pd), PC (1 mL), D_2_/CO (P_total_= 2.5 bar; 4:1) 100 °C. Yields were determined through GC-FID using mesitylene as internal standard. Data points are average values of 3 experiments and error bars represent standard deviations.

**Figure S7.** Insitu reversibility experiment by alternating feedgas and adding fresh substrate to the system without washing the catalyst. Reaction conditions: Pd@SILP (50 mg), substrate (1 mmol, 100 equiv. w.r.t to Pd), PC (1.5 mL), H_2_ (2 bar) or H_2_/CO (P_total_ = 2.5 bar; 4/1) 100 °C, 2 h. Conversion and yield were determined through NMR using mesitylene as internal standard. Substrate denoted in dark grey.

**Figure S8.** Catalyst recyclability using H_2_ and H_2_/CO over six cycles. Reaction conditions: Pd@SILP (20 mg), substrate (1 mmol, 250 equiv. w.r.t to Pd), PC (1.5 mL), H_2_ (2 bar) or H_2_/CO (P_total_ = 2.5 bar; 4/1) 100 °C, 2 h. Conversion and yield were determined through GC-FID using mesitylene as internal standard.

**Figure S9.** STEM-HAADF images of Pd nanoparticles in Pd@SILP after catalysis using a) H_2_ and b) H_2_/CO.

**Figure S10**. Size distribution of Pd nanoparticles in Pd@SILP after six reversible runs. Average diameter = 7.2±2.0 nm.

**Figure S11.** High-resolution XPS spectra of Pd3d after six runs under a) H_2_ and b) H_2_/CO.

**Table S6.** Variation of reaction conditions in the hydrogenation of **11** using Pd@SILP.

| Entry | Cat.loading (mol%) | P (bar) | Time (h) | Conv. (%) | | Yield **11a** (%) | Yield **11c** (%) |
| --- | --- | --- | --- | --- | --- | --- | --- |
| 1 | 2 | 4 | 4 | | 100 | 97 (*Z*:*E* = 98:2) | 3 |
| 2 | 0.33 | 4 | 2 | | 64 | 64 (*Z*:*E* = 100:0) | - |
| 3 | 0.2 | 4 | 2 | | 29 | 29 (*Z*:*E* = 100:0) | - |
| 4 | 0.2 | 2 | 2 | | 75 | 75 (*Z*:*E* = 100:0) | - |
| 5 | 0.2 | 1 | 2 | | 79 | 79 (*Z*:*E* = 99:1) | - |
| 6 | 0.2 | 1 | 6 | | 100 | 93 (*Z*:*E* = 99:1) | 7 |
| 7 | 0.2 | 2 | 6 | | 100 | 98 (*Z*:*E* = 99:1) | 2 |

Reaction Condition: **11** (1 mmol), Pd@SILP (w.r.t. **11**), PC (1 mL), H_2_/CO (P_total_= x bar; 99:1) 100 °C. Conversion and yield were determined through GC-FID using mesitylene as internal standard.

**Table** **S7.** Full product distribution of internal alkynes under H_2_/CO conditions

**Table** **S8.** Full product distribution of internal alkynes under H_2_ conditions

# 6. Analytical data for substrates

**methyl-3-(4-nitrophenyl)propiolate (5)** was obtained by following the general procedure on a 5 mmol scale and isolated by flash column chromatography (EtOAc:pentane = 1:4) as yellow solid. NMR data of the compound is in good agreement with the literature data.^[5]^

|  | **Formula:** C_10_H_7_NO_4_  **Molecular weight:** 205.17  **Isolated yield:** 0.35 g (34%)  **^1^H NMR (400 MHz, CDCl_3_)** δ 8.25 (d, *J = 8.6* Hz, 2H), 7.74 (d, *J = 8.9* Hz, 2H), 3.87 (s, 3H).  **^13^C NMR (101 MHz, CDCl_3_)** δ 153.86, 148.68, 133.85, 126.32, 123.91, 83.98, 83.27, 53.31. |
| --- | --- |

**1-fluoro-4-(phenylethynyl)benzene (12)** was obtained by following the general procedure on a 6 mmol scale and isolated by flash column chromatography (pentane) as a white solid. NMR data of the compound is in good agreement with the literature data.^[7]^

|  | **Formula:** C_14_H_9_F  **Molecular weight:** 196.22  **Isolated yield:** 1.09 g (86%)  **^1^H NMR (400 MHz, CDCl_3_)** δ 7.55-7.50 (m, 4H), 7.38-7.32 (m, 3H), 7.05 (t, *J = 8.7* Hz, 2H)  **^13^C NMR (101 MHz, CDCl_3_)** δ 162.6 (d, *J =* 198 Hz), 133.6 (d, *J =* 7 Hz), 131.7, 128.52, 128.48 123.2, 119.5, 115.8 (*J =* 18 Hz), 89.17, 88.4.  **^19^F NMR (376 MHz, CDCl_3_)** δ -110.98. |
| --- | --- |

**1-chloro-4-(phenylethynyl)benzene (13)** was obtained by following the general procedure on a 6 mmol scale and isolated by flash column chromatography (pentane) as a white solid. NMR data of the compound is in good agreement with the literature data.^[7]^

|  | **Formula:** C_14_H_9_Cl  **Molecular weight:** 212.68  **Isolated yield:** 0.75 g (59%)  **^1^H NMR (400 MHz, CDCl_3_)** δ 7.55-7.50 (m, 2H), 7.48-7.44 (m, 2H), 7.37-7.31 (m, 5H)  **^13^C NMR (101 MHz, CDCl_3_)** δ 134.41, 132.96, 131.75, 128.84, 128.63, 128.54, 123.09, 121.95, 90.46, 88.38. |
| --- | --- |

**1-(tert-butyl)-4-(phenylethynyl)benzene (15)** was obtained by following the general procedure on a 5 mmol scale and isolated by flash column chromatography (EtOAc:pentane = 2:98) as as a white solid. NMR data of the compound is in good agreement with the literature data.^[8]^

|  | **Formula:** C_18_H_18_  **Molecular weight:** 234.34  **Isolated yield:** 1.02 g (78%)  **^1^H NMR (400 MHz, CDCl_3_)** δ 7.56-7.51 (m, 2H), 7.48 (d, *J =* 8.4 Hz, 2H), 7.40-7.31 (m, 5H), 1.33 (s, 9H)  **^13^C NMR (101 MHz, CDCl_3_)** δ 151.67, 131.72, 131.47, 128.45, 128.21, 125.50, 123.65, 120.37, 34.94, 31.33. |
| --- | --- |

**1-(phenylethynyl)-4-(trifluoromethyl)benzene (16)** was obtained by following the general procedure on a 5 mmol scale and isolated by flash column chromatography (EtOAc:pentane = 2:98) as a white solid. NMR data of the compound is in good agreement with the literature data.^[7]^

|  | **Formula:** C_15_H_9_F_3_  **Molecular weight:** 246.23  **Isolated yield:** 1.2 g (97%)  **^1^H NMR (400 MHz, CDCl_3_)** δ 7.65-7.59 (m, 4H), 7.56-7.53 (m, 2H), 7.40-7.35 (m, 3H).  **^13^C NMR (101 MHz, CDCl_3_)** δ 131.95, 131.89, 130.1 (q, *J =* 33 Hz), 129.0, 128.6, 127.3, 125.4 (q, *J =* 4 Hz),124.1 (q, *J =* 270 Hz), 122.7, 91.9, 88.1.  19F NMR (MHz, CDCl3): -62.75. |
| --- | --- |

**1-(4-(phenylethynyl)phenyl)ethan-1-one (17)** was obtained by following the general procedure on a 5 mmol scale and isolated by flash column chromatography (EtOAc:pentane = 2:98) as a white solid. NMR data of the compound is in good agreement with the literature data.^[8]^

|  | **Formula:** C_16_H_12_O  **Molecular weight:** 220.27  **Isolated yield:** 0.95 g (86%)  **^1^H NMR (400 MHz, CDCl_3_)** δ 7.94 (d, *J =* Hz, 2H), 7.61 (d, *J =* Hz, 2H), 7.58-7.53 (m, 2H), 7.39-7.34 (m, 3H), 2.62 (s, 3H).  **^13^C NMR (101 MHz, CDCl_3_)** δ 197.50, 136.33, 131.89, 131.85, 128.97, 128.60, 128.43, 128.35, 122.79, 92.86, 88.74, 26.80. |
| --- | --- |

**1-nitro-4-(phenylethynyl)benzene (18)** was obtained by following the general procedure on a 5 mmol scale and isolated by flash column chromatography (EtOAc:pentane = 1:9) as a yellow solid. NMR data of the compound is in good agreement with the literature data.^[7]^

|  | **Formula:** C_16_H_12_NO_2_  **Molecular weight:** 223.23  **Isolated yield:** 1.02 g (90%)  **^1^H NMR (400 MHz, CDCl_3_)** δ 8.23 (d, *J =* 8.6 Hz, 2H), 7.67 (d, *J* = 8.9 Hz, 2H), 7.59-7.54 (m, 2H), 7.42-7.37 (m, 3H).  **^13^C NMR (101 MHz, CDCl_3_)** δ 147.12, 132.42, 131.99, 130.41, 129.43, 128.69, 123.79, 122.24, 94.85, 87.69. |
| --- | --- |

**4-(phenylethynyl)benzaldehyde (19)** was obtained by following the general procedure on a 5 mmol scale and isolated by flash column chromatography (EtOAc:pentane = 1:9) as an off-white solid. NMR data of the compound is in good agreement with the literature data.^[9]^

|  | **Formula:** C_15_H_10_O  **Molecular weight:** 206.24  **Isolated yield:** 0.76 g (74%)  **^1^H NMR (400 MHz, CDCl_3_)** δ 10.02 (s, 1H), 7.87 (d, *J =* 8.5 Hz, 2H), 7.68 (d, *J =* 8.2 Hz, 2H), 7.58-7.54 (m, 2H), 7.40-7.36 (m, 3H).  **^13^C NMR (101 MHz, CDCl_3_)** δ 191.55, 135.58, 132.26, 131.94, 129.78, 129.73, 129.12, 128.63, 122.66, 93.60, 88.66, 77.48, 77.36, 77.16, 76.84. |
| --- | --- |

**2-(phenylethynyl)thiophene (20)** was obtained by following the general procedure on a 5 mmol scale and isolated by flash column chromatography (EtOAc:pentane = 1:9) as an off-white solid. NMR data of the compound is in good agreement with the literature data.^[7]^

|  | **Formula:** C_12_H_8_S  **Molecular weight:** 184.03  **Isolated yield:** 0.76 g (82%)  **^1^H NMR (400 MHz, CDCl_3_)** δ 7.56-7.49 (m, 2H), 7.40-7.272 (m, 5H), 7.02 (t, *J* = 4.4 Hz, 1H)  **^13^C NMR (101 MHz, CDCl_3_)** δ 132.64, 132.03, 131.55, 129.36, 128.59, 128.56, 128.51, 127.39, 127.24, 123.45, 123.05, 93.16. |
| --- | --- |

**methyl(4-(phenylethynyl)phenyl)sulfane (21)** was obtained by following the general procedure on a 6 mmol scale and isolated by flash column chromatography (EtOAc:pentane = 5:95) as a pale yellow solid. NMR data of the compound is in good agreement with the literature data.^[7]^

|  | **Formula:** C_15_H_12_S  **Molecular weight:** 224.23  **Isolated yield:** 1.3 g (97%)  **^1^H NMR (400 MHz, CDCl_3_)** δ 7.55-7.49 (m, 2H), 7.44 (d, *J =* 8.4 Hz, 2H), 7.38-7.31 (m, 3H), 7.21 (d, *J =* 8.6 Hz, 2H), 2.50 (s, 3H)  **^13^C NMR (101 MHz, CDCl_3_)** δ 139.44, 132.02, 131.68, 128.49, 128.34, 126.01, 123.43, 119.69, 89.59, 89.32, 15.54. |
| --- | --- |

**1,3-dimethoxy-5-((4-methoxyphenyl)ethynyl)benzene (35)** was obtained by following the general procedure on a 10 mmol scale and isolated by flash column chromatography (EtOAc:pentane = 1:9) as a pale yellow oil. NMR data of the compound is in good agreement with the literature data.^[8]^

|  | **Formula:** C_17_H_16_O_3_  **Molecular weight:** 268.31  **Isolated yield:** 2.12 g (79%)  **^1^H NMR (400 MHz, CDCl_3_)** δ 7.47 (d, *J = 9.0* Hz, 2H), 6.88 (d, *J = 9.0* Hz, 2H), 6.68 (s, 2H), 6.45 (s, 1H), 3.83 (s, 3H), 3.80 (s, 6H)  **^13^C NMR (101 MHz, CDCl_3_)** δ 160.64, 159.80, 133.23, 124.99, 115.28, 114.12, 109.30, 101.65, 89.11, 88.20, 55.53, 55.41. |
| --- | --- |

**1-benzhydryl-4-(3-phenylprop-2-yn-1-yl)piperazine (36)** was obtained by following the general procedure on a 5 mmol scale and isolated by flash column chromatography (EtOAc:pentane = 2:8) as a yellow solid. NMR data of the compound is in good agreement with the literature data.^[6]^

|  | **Formula:** C_26_H_26_N_2_  **Molecular weight:** 366.51  **Isolated yield:** 1.5 g (85%)  **^1^H NMR (400 MHz, CDCl_3_)** δ 7.45-7.36 (m, 6H), 7.29-7.18 (m, 7H), 7.13 (t, *J = 7.3* Hz, 2H), 4.19 (s, 1H), 3.46 (s, 2H), 2.63 (brs, 4H), 2.44 (brs, 4H)  **^13^C NMR (101 MHz, CDCl_3_)** δ 142.89, 131.89, 128.61, 128.38, 128.20, 128.07, 127.05, 123.34, 85.32, 84.79, 76.36, 52.53, 51.99, 47.75. |
| --- | --- |

**N-methyl-N-(naphthalen-1-ylmethyl)-3-phenylprop-2-yn-1-amine (37)** was obtained by following the general procedure on a 5 mmol scale and isolated by flash column chromatography (EtOAc:pentane = 5:95) as a colourless oil. NMR data of the compound is in good agreement with the literature data.^[6]^

|  | **Formula:** C_21_H_19_N  **Molecular weight:** 285.39  **Isolated yield:** 1.25 g (88%)  **^1^H NMR (400 MHz, CDCl_3_)** δ 8.36 (d, *J = 7.3* Hz, 1H), 7.87 (d, *J = 7.3* Hz, 1H), 7.81 (d, *J = 7.3* Hz, 1H), 7.59-7.49 (m, 5H), 7.44 (d, *J = 7.3* Hz, 1H),7.38-7.33 (m, 3H), 4.09 (s, 2H), 3.58 (s, 2H), 2.49 (s, 3H).**^13^C NMR (101 MHz, CDCl_3_)** δ 134.41, 134.02, 132.74, 131.88, 128.56, 128.46, 128.33, 128.18, 127.87, 126.15, 125.77, 125.27, 124.79, 123.49, 86.05, 84.68, 58.37, 46.01, 42.34. |
| --- | --- |

# 17. Analytical data for Isolated products

**(Z)-4-phenylbut-3-en-2-one (1a)** was obtained by following the general procedure on a 1 mmol scale and isolated by flash column chromatography (EtOAc:pentane = 1:4) as pale yellow oil. NMR data of the compound is in good agreement with the literature data.^[8]^

|  | **Formula:** C_10_H_10_O  **Molecular weight:** 146.16  **Isolated yield:** 911 mg (78%)  **^1^H NMR (400 MHz, C_6_D_6_)** δ 7.54 (d, *J =* 7.7 Hz, 2H), 7.18-7.08 (m, 3H), 6.49 (d, *J =* 13.0 Hz, 1H), 5.90 (d, *J =* 13.0 Hz, 1H), 1.87 (s, 3H).  **^13^C NMR (101 MHz, C_6_D_6_)** δ 198.9, 139.3, 135.9, 123.0, 129.5, 129.2, 128.5, 30.6. |
| --- | --- |

**(Z)-3-phenylacrylic acid (2a)** was obtained by following the general procedure on a 1 mmol scale and isolated by flash column chromatography (EtOAc:pentane = 1:4) as colorless liquid. NMR data of the compound is in good agreement with the literature data.^[8]^

|  | **Formula:** C_9_H_8_O_2_  **Molecular weight:** 148.16  **Isolated yield: 136 mg (92%)**  **^1^H NMR (400 MHz, CDCl_3_)** δ 7.63-7.58 (m, 2H), 7.38-7.33 (m, 3H), 7.07 (d, *J =* 12.6 Hz, 1H), 5.97 (d, *J =* 12.6 Hz, 1H).  **^13^C NMR (101 MHz, CDCl_3_)** δ 171.48, 145.99, 134.51, 130.10, 129.53, 128.24, 118.82. |
| --- | --- |

**3-phenylpropanoic acid (2c)** was obtained by following the general procedure on a 1 mmol scale and isolated by flash column chromatography (EtOAc:pentane = 1:9) as a white solid. NMR data of the compound is in good agreement with the literature data.^[10]^

|  | **Formula:** C_9_H_10_O_2_  **Molecular weight:** 150.18  **Isolated yield:** 141 mg (94%)  **^1^H NMR (400 MHz, CDCl_3_)** δ 7.31 (t, *J =* 7.8 Hz, 2H), 7.17-7.11 (m, 3H), 2.98 (t, *J =* 7.9 Hz, 2H), 2.70 (t, *J =* 7.9 Hz, 2H)  **^13^C NMR (101 MHz, CDCl_3_)** δ 179.37, 140.27, 128.70, 128.40, 126.52, 35.75, 30.69. |
| --- | --- |

**(Z)-3-phenylprop-2-en-1-ol (6a)** was obtained by following the general procedure on a 1 mmol scale and isolated by flash column chromatography (EtOAc:pentane = 1:4) as colorless oil. NMR data of the compound is in good agreement with the literature data.^[8]^

|  | **Formula:** C_9_H_10_O  **Molecular weight:** 134.19  **Isolated yield:122 mg (91%)**  **^1^H NMR (400 MHz, CDCl_3_)** δ 7.28-7.07 (m, 5H), 6.46 (d, *J =* 11.7 Hz, 1H), 5.77 (dt, *J =* 11.7 Hz, 6.4 Hz, 1H), 4.33 (dd, *J =* 6.4 Hz, 1.8 Hz 2H), 1.97 (brs, 1H)  **^13^C NMR (101 MHz, CDCl_3_)** δ 136.61, 131.31, 130.97, 128.86, 128.35, 127.33, 59.71. |
| --- | --- |

**(Z)-1-chloro-4-styrylbenzene (13a)** was obtained by following the general procedure on a 1 mmol scale and isolated by flash column chromatography (EtOAc:pentane = 5:95) as a yellow oil. NMR data of the compound is in good agreement with the literature data.^[8]^

|  | **Formula:** C_14_H_11_Cl  **Molecular weight:** 214.69  **Isolated yield:**  **^1^H NMR (400 MHz, CDCl_3_)** δ 7.14-7.03 (m, 9H), 6.51 (d, *J =* 12.2 Hz, 1H), 6.41 (d, *J =* 12.2 Hz, 1H)  **^13^C NMR (101 MHz, CDCl_3_)** δ 136.96, 135.74, 132.85, 131.05, 130.33, 129.02, 128.91, 128.52, 128.48, 128.45, 127.43. |
| --- | --- |

**1-chloro-4-phenethylbenzene (13c)** was obtained by following the general procedure on a 1 mmol scale and isolated by flash column chromatography (EtOAc:pentane = 2:98) as colorless oil. NMR data of the compound is in good agreement with the literature data.^[11]^

|  | **Formula:** C_14_H_13_Cl  **Molecular weight:** 216.71  **Isolated yield:** 180 mg (83%)  **^1^H NMR (400 MHz, CDCl_3_)** δ 7.38-7.19 (m, 7H), 7.15 (d, *J =* 8.4 Hz, 2H), 2.96 (m, 4H)  **^13^C NMR (101 MHz, CDCl_3_)** δ 141.38, 140.22, 131.73, 129.96, 128.58, 128.51, 128.49, 128.45, 126.16, 37.87, 37.31. |
| --- | --- |

**(Z)-1-bromo-4-styrylbenzene (14a)** was obtained by following the general procedure on a 1 mmol scale and isolated by flash column chromatography (EtOAc:pentane = 5:95) as a yellow oil. NMR data of the compound is in good agreement with the literature data.^[8]^

|  | **Formula:** C_14_H_11_Br  **Molecular weight:** 259.19  **Isolated yield:** 231 mg (89%)  **^1^H NMR (400 MHz, CDCl_3_)** δ 7.38 (d, *J =* 8.3 Hz, 2H), 7.30-7.24 (m, 5H), 7.15 (d, *J =* 8.3 Hz, 2H), 6.68 (d, *J =* 12.2 Hz, 1H), 6.54 (d, *J =* 12.2 Hz, 1H)  **^13^C NMR (101 MHz, CDCl_3_)** δ 136.94, 136.21, 131.48, 131.15, 130.66, 129.06, 128.91, 128.48, 127.47, 121.06. |
| --- | --- |

**(Z)-1-(tert-butyl)-4-styrylbenzene (15a)** was obtained by following the general procedure on a 1 mmol scale and isolated by flash column chromatography (EtOAc:pentane = 2:98) as a white solid. NMR data of the compound is in good agreement with the literature data.^[8]^

|  | **Formula:** C_18_H_20_  **Molecular weight:** 236.36  **Isolated yield:** 206 mg (87%)  **^1^H NMR (400 MHz, CDCl_3_)** δ 7.31-7.18 (m, 9H), 6.55 (s, 2H), 1.29 (s, 9H)  **^13^C NMR (101 MHz, CDCl_3_)** δ 150.27, 137.73, 134.32, 130.22, 129.71, 128.95, 128.72, 128.35, 127.10, 125.21, 34.67, 31.42. |
| --- | --- |
|  |  |

**1-phenethyl-4-(trifluoromethyl)benzene (16c)** was obtained by following the general procedure on a 1 mmol scale and isolated by flash column chromatography (EtOAc:pentane = 2:98) as pale yellow oil. NMR data of the compound is in good agreement with the literature data.^[11]^

|  | **Formula:** C_15_H_13_F_3_  **Molecular weight:** 250.26  **Isolated yield:** 225 mg (90%)  **^1^H NMR (400 MHz, CDCl_3_)** δ 7.43 (d, *J =* 8.1 Hz, 2H), 7.23-7.04 (m, 7H). 2.91-2.79 (m, 4H)  **^13^C NMR (101 MHz, CDCl_3_)** δ145.80, 141.08, 128.84, 128.48 (s, 4C, *ortho/meta* overlap), 128.35 (q, *J* = 32 Hz) 126.2, 125.27 (q, *J =* 3.7 Hz), 124.4 (q, *J =* 272 Hz) 37.10, 37.55.  **^19^F NMR (376 MHz, CDCl_3_):** δ -62.23. |
| --- | --- |

**(Z)-1-(4-styrylphenyl)ethan-1-one (17a)** was obtained by following the general procedure on a 1 mmol scale and isolated by flash column chromatography (EtOAc:pentane = 5:95) as colorless oil. NMR data of the compound is in good agreement with the literature data.^[8]^

|  | **Formula:** C_16_H_14_O  **Molecular weight:** 222.29  **Isolated yield:** 189 mg (85%)  **^1^H NMR (400 MHz, CDCl_3_)** δ 7.82 (d, *J =* 8.4 Hz, 2H), 7.33 (d, *J =* 8.3 Hz, 2H), 6.73 (d, *J =* 8.3 Hz, 2H), 6.61 (d, *J =* 12.3 Hz, 2H), 2.57 (s, 3H).  **^13^C NMR (101 MHz, CDCl_3_)** δ 197.75, 142.39, 136.76, 135.70, 132.54, 129.22, 129.15, 128.95, 128.48, 128.43, 127.67, 26.67. |
| --- | --- |

**(Z)-1-nitro-4-styrylbenzene (18a)** was obtained by following the general procedure on a 1 mmol scale and isolated by flash column chromatography (EtOAc:pentane = 1:4) as yellow solid. NMR data of the compound is in good agreement with the literature data.^[12]^

|  | **Formula:** C_14_H_11_NO_2_  **Molecular weight:** 225.25  **Isolated yield:** 191 mg (85%)  **^1^H NMR (400 MHz, CDCl_3_)** δ 8.07 (d, *J =* 8.7 Hz, 2H), 7.37 (d, *J =* 8.7 Hz, 2H), 7.28-7.24 (m, 4H), 7.22-7.18 (m, 2H), 6.82 (d, *J =* 12.3 Hz, 1H), 6.62 (d, *J =* 12.3 Hz, 1H)  **^13^C NMR (101 MHz, CDCl_3_)** δ 144.31, 136.25, 134.09, 129.80, 128.94, 128.71, 128.14, 128.10, 123.72, 77.48. |
| --- | --- |

**(Z)-4-styrylbenzaldehyde (19a)** was obtained by following the general procedure on a 1 mmol scale and isolated by flash column chromatography (EtOAc:pentane = 1:4) as yellow solid. NMR data of the compound is in good agreement with the literature data.^[13]^

|  | **Formula:** C_15_H_12_O  **Molecular weight:** 208.26  **Isolated yield: 179 mg (86%)**  **^1^H NMR (400 MHz, CDCl_3_)** δ 9.95 (s, 1H), 7.73 (d, *J =* 8.4 Hz, 2H), 7.40 (d, *J =* 8.4 Hz, 2H), 7.27-7.20 (m, 5H), 6.77 (d, *J =* 12.3 Hz, 2H), 6.62 (d, *J =* 12.3 Hz, 2H)  **^13^C NMR (101 MHz, CDCl_3_)** δ 191.88, 143.86, 136.60, 135.05, 133.09, 129.81, 129.60, 129.09, 128.96, 128.53, 127.80 |
| --- | --- |

**4-ethylaniline (28c)** was obtained by following the general procedure on a 1 mmol scale and isolated by flash column chromatography (EtOAc:pentane = 3:7) as a pale yellow oil. NMR data of the compound is in good agreement with the literature data.^[14]^

|  | **Formula:** C_8_H_11_N  **Molecular weight:** 121.18  **Isolated yield:** 98 mg (81%)  **^1^H NMR (400 MHz, CDCl_3_)** δ 6.99 (d, J *=* 8.3 Hz, 2H), 6.63 (d, *J =* 8.3 Hz, 2H), 3.53 (brs, 2H), 2.54 (q, *J =* 7.6 Hz, 2H), 1.19 (t, *J =* 7.6 Hz, 3H).  **^13^C NMR (101 MHz, CDCl_3_)** δ 144.17, 134.62, 128.73, 115.41, 28.12, 16.10. |
| --- | --- |

**3,7-dimethylocta-1,6-dien-3-ol (32a)** was obtained by following the general procedure on a 1 mmol scale and isolated by flash column chromatography (EtOAc:pentane = 2:98) as colorless liquid. NMR data of the compound is in good agreement with the literature data.^[15]^

|  | **Formula:** C_10_H_18_O  **Molecular weight:** 154.25  **Isolated yield: 142 mg (92%)**  **^1^H NMR (400 MHz, CDCl_3_)** δ 5.90 (dd, *J* = 17.4, 10.7 Hz, 1H), 5.21 (dd, *J* = 17.3, 1.3 Hz, 1H), 5.15-5.08 (m, 1H), 5.05 (dd, *J* = 10.8, 1.3 Hz, 1H), 2.10-1.93 (m, 1H), 1.70-1.51 (m, 9H), 1.27 (s, 3H).  **^13^C NMR (101 MHz, CDCl_3_)** δ 145.15, 132.08, 124.44, 111.81, 73.61, 42.17, 28.00, 25.83, 22.92, 17.83. |
| --- | --- |

**(8R,9S,10R,13S,14S,17R)-17-hydroxy-10,13-dimethyl-17-vinyl-1,2,6,7,8,9,10,11,12,13,14,15,16,17-tetradecahydro-3H-cyclopenta[a]phenanthren-3-one (33a)** was obtained by following the general procedure on a 1 mmol scale and isolated by evaporating the volatiles to afford **34a** as white solid. NMR data of the compound is in good agreement with the literature data.^[16]^

|  | **Formula:** C_21_H_30_O_2_  **Molecular weight:** 314.47  **Isolated yield: 298 mg (95%)**  **^1^H NMR (400 MHz, CDCl_3_)** δ 6.03 (dd, *J* = 17.6, 10.8 Hz, 1H), 5.72 (s, 1H), 5.19–5.08 (m, 2H), 2.47–2.23 (m, 4H), 2.07-1.91 (m, 2H), 1.90–1.84 (m, 2H), 1.72–1.47 (m, 7H), 1.46–1.32 (m, 2H), 1.30–1.16 (m, 5H), 1.07–0.82 (m, 5H).  **^13^C NMR (101 MHz, CDCl_3_)** δ 199.58, 171.43, 142.90, 123.71, 112.11, 83.80, 53.56, 49.29, 46.09, 38.57, 36.17, 35.79, 35.59, 33.86, 32.77, 31.85, 31.59, 23.46, 20.58, 17.34, 14.03. |
| --- | --- |

**(Z)-1,3-dimethoxy-5-(4-methoxystyryl)benzene (35a)** was obtained by following the general procedure on a 1 mmol scale and isolated by flash column chromatography (EtOAc:pentane = 5:95) as colorless oil. NMR data of the compound is in good agreement with the literature data.^[8]^

|  | **Formula:** C_17_H_18_O_3_  **Molecular weight:** 270.33  **Isolated yield:** 214 mg (79%)  **^1^H NMR (400 MHz, CDCl_3_)** δ 7.22 (d, *J =* 8.6 Hz, 2H), 6.77 (d, *J =* 8.6 Hz, 2H), 6.53 (*J =* 12.3 Hz, 1H), 6.47-6.43 (m, 3H), 6.33 (t, *J =* 2.3 Hz, 1H), 3.79 (s, 3H), 3.68 (s, 6H)  **^13^C NMR (101 MHz, CDCl_3_)** δ 160.68, 158.85, 139.60, 130.40, 130.28, 129.66, 128.79, 113.64, 106.72, 99.76, 55.33. |
| --- | --- |
|  |  |

**(Z)-N-methyl-N-(naphthalen-1-ylmethyl)-3-phenylprop-2-en-1-amine (37a)** was obtained by following the general procedure on a 1 mmol scale and isolated by flash column chromatography (EtOAc:pentane = 1:9) as colorless oil. NMR data of the compound is in good agreement with the literature data.^[6]^

|  | **Formula:** C_21_H_21_N  **Molecular weight:** 287.41  **Isolated yield:** 233 (81%)  **^1^H NMR (400 MHz, CDCl_3_)** δ 8.33 (d, *J =* 8.7 Hz, 1H), 7.86 (d, *J =* 7.8 Hz, 1H), 7.80 (d, *J =* 7.8 Hz, 1H), 7.54-7.46 (m,2H), 7.39-7.36 (m, 2H), 7.27-7.24 (m, 5H), 6.62 (d, *J =* 12 Hz, 1H), 5.95 (dt, *J =* 12 Hz, 6.4 Hz 1H), 3.91 (s, 2H), 3.39 (dd, *J =* 6.8 Hz, 1.9 Hz 1H), 2.26 (s, 3H).  **^13^C NMR (101 MHz, CDCl_3_)** δ 137.26, 134.87, 134.00, 132.65, 131.60, 130.29, 129.11, 128.55, 128.21, 128.08, 127.67, 126.95, 125.98, 125.72, 125.25, 124.84, 60.46, 55.74, 42.76. |
| --- | --- |

# 18. ^1^H and ^13^C NMR spectra of isolated substrates and products

**Figure S12.** ^1^H NMR (400 MHz, CDCl_3_) spectrum of crude reaction of **1** (1 mmol scale) to yield product **1a+1b** with mesitylene as internal standard (0.3 mmol)**.**

**Figure S13.** ^1^H NMR (400 MHz, CDCl_3_) spectrum of crude reaction of **24** (1 mmol scale) to yield product **24a** with mesitylene as internal standard (0.3 mmol)**.**

**Figure S14.** ^1^H NMR (400 MHz, CDCl_3_) spectrum of crude reaction of **34** (0.17 mmol scale) to yield product **34a** with mesitylene as internal standard (0.3 mmol)**.**

**Figure S15.** ^1^H NMR (400 MHz, CDCl_3_) spectrum of substrate **5**.

**Figure S16.** ^13^C NMR (101 MHz, CDCl_3_) spectrum of substrate **5.**

**Figure S17.** ^1^H NMR (400 MHz, CDCl_3_) spectrum of substrate **12**.

**Figure S18.** ^13^C NMR (101 MHz, CDCl_3_) spectrum of substrate **12**.

**Figure S19.** ^19^F NMR (376 MHz, CDCl_3_) spectrum of substrate **12**.

**Figure S20.** ^1^H NMR (400 MHz, CDCl_3_) spectrum of substrate **13**.

**Figure S21.** ^13^C NMR (101 MHz, CDCl_3_) spectrum of substrate **13**.

**Figure S22.** ^1^H NMR (400 MHz, CDCl_3_) spectrum of substrate **15**.

**Figure S23.** ^13^C NMR (101 MHz, CDCl_3_) spectrum of substrate **15**.

**Figure S24.** ^1^H NMR (400 MHz, CDCl_3_) spectrum of substrate **16**.

**Figure S25.** ^13^C NMR (101 MHz, CDCl_3_) spectrum of substrate **16**.

**Figure S26.** ^19^F NMR (376 MHz, CDCl_3_) spectrum of substrate **16**.

**Figure S27.** ^1^H NMR (400 MHz, CDCl_3_) spectrum of substrate **17**.

**Figure S28.** ^13^C NMR (101 MHz, CDCl_3_) spectrum of substrate **17**.

**Figure S29.** ^1^H NMR (400 MHz, CDCl_3_) spectrum of substrate **18**.

**Figure S30.** ^13^C NMR (101 MHz, CDCl_3_) spectrum of substrate **18**.

**Figure S31.** ^1^H NMR (400 MHz, CDCl_3_) spectrum of substrate **19**.

**Figure S32.** ^13^C NMR (101 MHz, CDCl_3_) spectrum of substrate **19**.

**Figure S33.** ^1^H NMR (400 MHz, CDCl_3_) spectrum of substrate **20**.

**Figure S34.** ^13^C NMR (101 MHz, CDCl_3_) spectrum of substrate **20**.

**Figure S35.** ^1^H NMR (400 MHz, CDCl_3_) spectrum of substrate **21**.

**Figure S36.** ^13^C NMR (101 MHz, CDCl_3_) spectrum of substrate **21**.

**Figure S37.** ^1^H NMR (400 MHz, CDCl_3_) spectrum of substrate **35**.

**Figure S38.** ^13^C NMR (101 MHz, CDCl_3_) spectrum of substrate **35**.

**Figure S39.** ^1^H NMR (400 MHz, CDCl_3_) spectrum of substrate **36**.

**Figure S40.** ^13^C NMR (101 MHz, CDCl_3_) spectrum of substrate **36**.

**Figure S41.** ^1^H NMR (400 MHz, CDCl_3_) spectrum of substrate **37**.

**Figure S42.** ^13^C NMR (101 MHz, CDCl_3_) spectrum of substrate **37**.

**Figure S43.** ^1^H NMR (400 MHz, C_6_D_6_) spectrum of product **1a**.

**Figure S44.** ^13^C NMR (101 MHz, C_6_D_6_) spectrum of product **1a**.

**Figure S45.** ^1^H NMR (400 MHz, CDCl_3_) spectrum of product **2a**.

**Figure S46.** ^13^C NMR (101 MHz, CDCl_3_) spectrum of product **2a**.

**Figure S47.** ^1^H NMR (400 MHz, CDCl_3_) spectrum of product **2c**.

**Figure S48.** ^13^C NMR (101 MHz, CDCl_3_) spectrum of substrate **2c**.

**Figure S49.** ^1^H NMR (400 MHz, CDCl_3_) spectrum of product **6a**.

**Figure S50.** ^13^C NMR (101 MHz, CDCl_3_) spectrum of product **6a**.

**Figure S51.** ^1^H NMR (400 MHz, CDCl_3_) spectrum of product **13a**.

**Figure S52.** ^13^C NMR (101 MHz, CDCl_3_) spectrum of product **13a**.

**Figure S53.** ^1^H NMR (400 MHz, CDCl_3_) spectrum of product **13c**.

**Figure S54.** ^13^C NMR (101 MHz, CDCl_3_) spectrum of product **13c**.

**Figure S55.** ^1^H NMR (400 MHz, CDCl_3_) spectrum of product **14a**.

**Figure S56.** ^13^C NMR (101 MHz, CDCl_3_) spectrum of product **14a**.

**Figure S57.** ^1^H NMR (400 MHz, CDCl_3_) spectrum of product **15a**.

**Figure S58.** ^13^C NMR (101 MHz, CDCl_3_) spectrum of product **15a**.

**Figure S59.** ^1^H NMR (400 MHz, CDCl_3_) spectrum of product **16c**.

**Figure S60.** ^13^C NMR (101 MHz, CDCl_3_) spectrum of product 1**6c**.

**Figure S61.** ^19^F NMR (376 MHz, CDCl_3_) spectrum of product **16c**.

**Figure S62.** ^1^H NMR (400 MHz, CDCl_3_) spectrum of product **17a**.

**Figure S63.** ^13^C NMR (101 MHz, CDCl_3_) spectrum of product 1**7a**.

**Figure S64.** ^1^H NMR (400 MHz, CDCl_3_) spectrum of product **18a**.

**Figure S65.** ^13^C NMR (101 MHz, CDCl_3_) spectrum of product 1**8a**.

**Figure S66.** ^1^H NMR (400 MHz, CDCl_3_) spectrum of product **19a**.

**Figure S67.** ^13^C NMR (101 MHz, CDCl_3_) spectrum of product 1**9a**.

**Figure S68.** ^1^H NMR (400 MHz, CDCl_3_) spectrum of product **28c**.

**Figure S69.** ^13^C NMR (101 MHz, CDCl_3_) spectrum of product **28c**.

**Figure S70.** ^1^H NMR (400 MHz, CDCl_3_) spectrum of product **32a**.

**Figure S71.** ^13^C NMR (101 MHz, CDCl_3_) spectrum of product **32a**.

**Figure S72.** ^1^H NMR (400 MHz, CDCl_3_) spectrum of product **33a**.

**Figure S73.** ^13^C NMR (101 MHz, CDCl_3_) spectrum of product **33a**.

**Figure S74.** ^1^H NMR (400 MHz, CDCl_3_) spectrum of product **35a**.

**Figure S75.** ^13^C NMR (101 MHz, CDCl_3_) spectrum of product **35a**.

**Figure S76.** ^1^H NMR (400 MHz, CDCl_3_) spectrum of product **37a**.

**Figure S77.** ^13^C NMR (101 MHz, CDCl_3_) spectrum of product **37a**.

# 19. References

[1]	K.L. Luska, A. Bordet, S. Tricard, I. Sinev, W. Grünert, B. Chaudret, W. Leitner, “Enhancing the Catalytic Properties of Ruthenium Nanoparticle-SILP Catalysts by Dilution with Iron,” *ACS Catalysis* **6** (2016): 3719-3726, <https://doi.org/10.1021/acscatal.6b00796>.

[2]	J. Hu, L.M. Lacroix, J. Johny, S. Ghosh, E.H. Wolf, J. Ji, S.H. Lin, M. Durai, A.B. Schone, W. Hetaba, H. Ruland, W. Leitner, A. Bordet, “Low-Temperature Reverse Water-Gas Shift Enabled by Magnetically Induced Catalysis,” *Angewandte Chemie International Edition* **n/a** (2026): e23576, <https://doi.org/10.1002/anie.202523576>.

[3]	G. Fernández, J. Sort, R. Pleixats, “Nickel Nanoparticles Stabilized by Trisimidazolium Salts: Synthesis, Characterization and Application as Recyclable Catalysts for the Reduction of Nitroarenes,” *ChemistrySelect* **3** (2018): 8597-8603, <https://doi.org/10.1002/slct.201801839>.

[4]	S.H. Lin, W. Hetaba, B. Chaudret, W. Leitner, A. Bordet, “Copper-Decorated Iron Carbide Nanoparticles Heated by Magnetic Induction as Adaptive Multifunctional Catalysts for the Selective Hydrodeoxygenation of Aldehydes,” *Advanced Energy Materials* **12** (2022): 2201783, <https://doi.org/https://doi.org/10.1002/aenm.202201783>.

[5]	B. Keen, C. Cong, A. Castanedo, G.H.M. Davies, R.R. Knowles, H.M.L. Davies, “Stereoselective Synthesis of Highly Functionalized Bicyclo[2.1.0]pentanes by Sequential [2 + 1] and [2 + 2] Cycloadditions,” *Organic Letters* **27** (2025): 1673-1678, <https://doi.org/10.1021/acs.orglett.5c00054>.

[6]	D. Kalsi, S.J.L. Anandaraj, M. Durai, C. Weidenthaler, M. Emondts, S.P. Nolan, A. Bordet, W. Leitner, “One-Pot Multicomponent Synthesis of Allyl and Alkylamines Using a Catalytic System Composed of Ruthenium Nanoparticles on Copper N-Heterocyclic Carbene-Modified Silica,” *ACS Catalysis* **12** (2022): 14902-14910, <https://doi.org/10.1021/acscatal.2c04044>.

[7]	R. Kusy, M. Lindner, J. Wagner, K. Grela, “Ligand-to-metal ratio controls stereoselectivity: Highly functional-group- tolerant, iridium-based, (*E*)- selective alkyne transfer semihydrogenation,” *Chem Catalysis* **2** (2022): 1346-1361, <https://doi.org/10.1016/j.checat.2022.04.014>.

[8]	V. Chugh, J.J. Wu, M. Leutzsch, H. Randel, T. Weyhermüller, A.A. Auer, C. Farès, C. Werlé, “Controlling hydrogen transfer dynamics in adaptive semihydrogenation of alkynes: Unveiling and directing outer- vs. inner-sphere mechanisms,” *Chem Catalysis* **4** (2024): 101078, <https://doi.org/10.1016/j.checat.2024.101078>.

[9]	B.A. Martek, M. Gazvoda, D. Urankar, J. Kosmrlj, “Designing Homogeneous Copper-Free Sonogashira Reaction through a Prism of Pd-Pd Transmetalation,” *Organic Letters* **22** (2020): 4938-4943, <https://doi.org/10.1021/acs.orglett.0c01227>.

[10]	X. Jiang, J. Zhang, S. Ma, “Iron Catalysis for Room-Temperature Aerobic Oxidation of Alcohols to Carboxylic Acids,” *Journal of the American Chemical Society* **138** (2016): 8344-8347, <https://doi.org/10.1021/jacs.6b03948>.

[11]	F. Zhou, X. Hu, W. Zhang, C.J. Li, “Copper-Catalyzed Radical Reductive Arylation of Styrenes with Aryl Iodides Mediated by Zinc in Water,” *The Journal of Organic Chemistry* **83** (2018): 7416-7422, <https://doi.org/10.1021/acs.joc.8b00278>.

[12]	H. Zhao, Y. Wang, J.C. Sha, S.R. Sheng, M.Z. Cai, “MCM-41-supported bidentate phosphine palladium(0) complex as an efficient catalyst for the heterogeneous Stille reaction,” *Tetrahedron* **64** (2008): 7517-7523, <https://doi.org/10.1016/j.tet.2008.05.120>.

[13]	J.B. Xu, N. Liu, H.P. Lv, C.X. He, Z.N. Liu, X.F. Shen, F.X. Cheng, B.M. Fan, “Photocatalyst-free visible light promoted *E*→*Z* isomerization of alkenes,” *Green Chemistry* **22** (2020): 2739-2743, <https://doi.org/10.1039/c9gc04303a>.

[14]	Y.H. Sun, S.H. Zhang, M.Z. Cai, P.T. Ma, J.Y. Niu, J.P. Wang, “Inorganic ligand-protected synthesis and characterization of a {Ag_6_} cluster within an annular polyoxometalate,” *Journal of Materials Chemistry A* **12** (2024): 29580-29587, <https://doi.org/10.1039/d4ta05238b>.

[15]	A. Guzman-Martinez, A.H. Hoveyda, “Enantioselective synthesis of allylboronates bearing a tertiary or quaternary B-substituted stereogenic carbon by NHC-Cu-catalyzed substitution reactions,” *Journal of American Chemical Society* **132** (2010): 10634-10637, <https://doi.org/10.1021/ja104254d>.

[16]	S. Mori, T. Ohkubo, T. Ikawa, A. Kume, T. Maegawa, Y. Monguchi, H. Sajiki, “Pd(0)-polyethyleneimine complex as a partial hydrogenation catalyst of alkynes to alkenes,” *Journal of Molecular Catalysis A: Chemical* **307** (2009): 77-87, <https://doi.org/10.1016/j.molcata.2009.03.013>.
